# Folic Acid–Peptide Conjugates Combine Selective Cancer Cell Internalization with Thymidylate Synthase Dimer Interface Targeting

**DOI:** 10.1021/acs.jmedchem.0c02107

**Published:** 2021-03-12

**Authors:** Gaetano Marverti, Chiara Marraccini, Andrea Martello, Domenico D’Arca, Salvatore Pacifico, Remo Guerrini, Francesca Spyrakis, Gaia Gozzi, Angela Lauriola, Matteo Santucci, Giuseppe Cannazza, Lorenzo Tagliazucchi, Addolorata Stefania Cazzato, Lorena Losi, Stefania Ferrari, Glauco Ponterini, Maria P. Costi

**Affiliations:** † Department Biomedical, Metabolic and Neural Sciences, University of Modena and Reggio Emilia, Via Campi 287, 41125 Modena, Italy; ‡ Department Life Sciences, University of Modena and Reggio Emilia, Via Campi 103, 41125 Modena, Italy; § Department of Chemical and Pharmaceutical Sciences, University of Ferrara, Via Fossato di Mortara, 44121 Ferrara, Italy

## Abstract

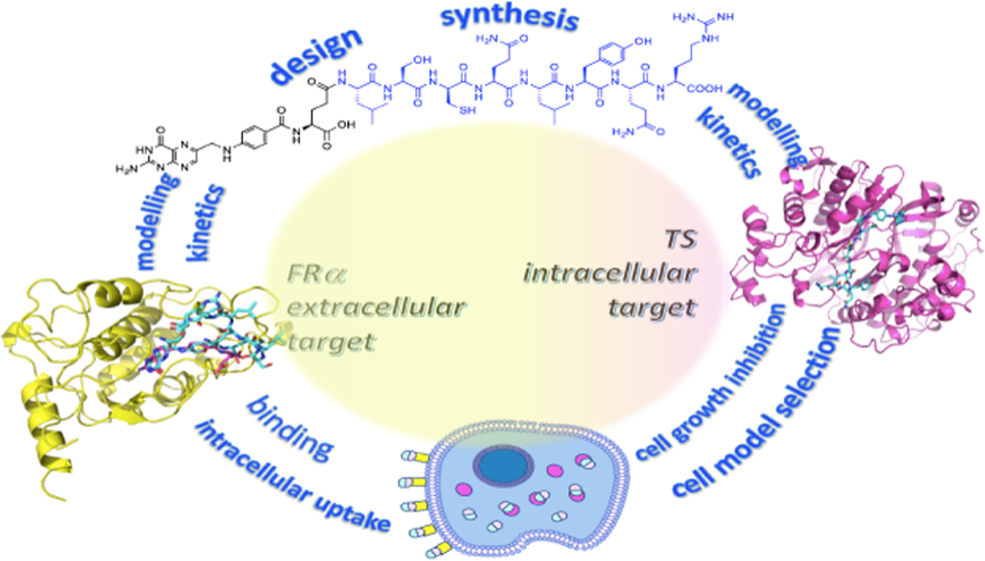

Drug–target interaction, cellular internalization, and target engagement should be addressed to design a lead with high chances of success in further optimization stages. Accordingly, we have designed conjugates of folic acid with anticancer peptides able to bind human thymidylate synthase (hTS) and enter cancer cells through folate receptor α (FRα) highly expressed by several cancer cells. Mechanistic analyses and molecular modeling simulations have shown that these conjugates bind the hTS monomer–monomer interface with affinities over 20 times larger than the enzyme active site. When tested on several cancer cell models, these conjugates exhibited FRα selectivity at nanomolar concentrations. A similar selectivity was observed when the conjugates were delivered in synergistic or additive combinations with anticancer agents. At variance with 5-fluorouracil and other anticancer drugs that target the hTS catalytic pocket, these conjugates do not induce overexpression of this protein and can thus help combating drug resistance associated with high hTS levels.

## Introduction

**Figure 1:**
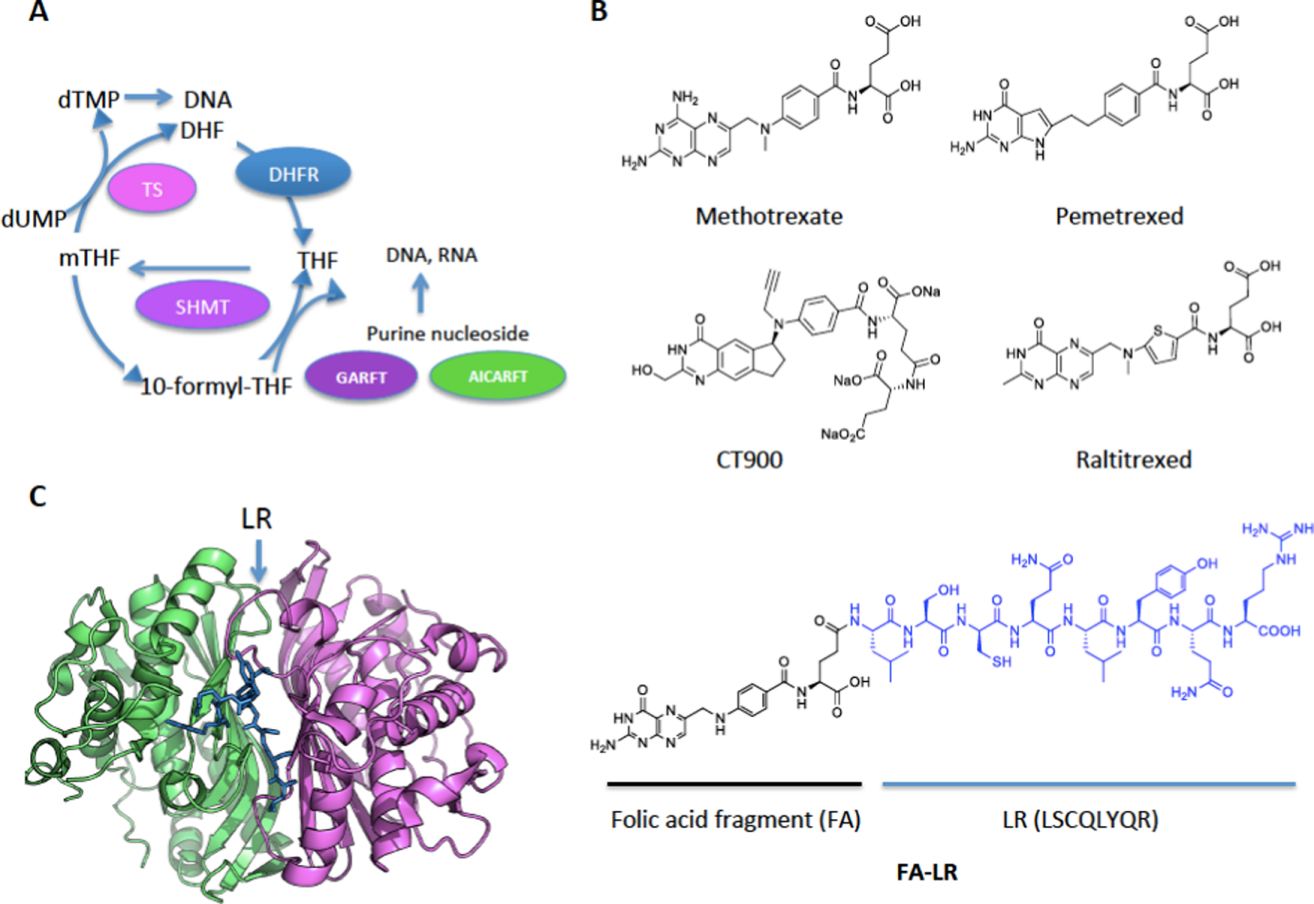
(A) hTS cycle with the folate enzymes involved and connection to DNA and purine nucleoside syntheses. TS, thymidylate synthase; DHFR, dihydrofolate reductase; SHMT, serine-hydroxymethyl transferase; GARFT, glycinamide ribonucleotide formyltransferase; AICARTF, aminoimidazolecarboxamide ribonucleotide formyltransferase. (B) Structures of folic acid (FA) and of several folate-analogue inhibitors of thymidylate synthase that enter cancer cells through the reduced folate carrier (RFC) (pemetrexed, methotrexate, raltitrexed) and FRα (CT900); FA–LR peptide conjugate. (C) Details of the X-ray crystal structure of hTS with the interface-bound LR peptide inhibitor.

A holistic approach to drug discovery takes into account the predictable multitarget interactions and focuses on both cellular internalization of the potential drug and its intracellular binding to on- and off-targets. To move in such direction, in this work, we have tracked the trafficking of two new anticancer lead compounds from the region outside cells to their intracellular target, i.e., the human thymidylate cycle^1,2^ that involves the enzymes thymidylate synthase (TS, EC:2.1.1.45), dihydrofolate reductase (DHFR, EC:1.5.1.3), and serine-hydroxymethyl transferase (SHMT, EC:2.1.2.1). All of the reactions catalyzed by these enzymes constitute essential steps in the biosynthesis of DNA nucleotide bases.^2,3^ Two additional enzymes are crucial to the purine nucleoside synthesis, namely, glycinamide ribonucleotide formyltransferase (GARFT, 2.1.2.2) and aminoimidazolecarboxamide ribonucleotide formyltransferase (AICARTF, 3.5.4.10) (Figure 1A). The methylation reaction catalyzed by human thymidylate synthase (hTS) provides the only cellular source of 2′-deoxythimidine monophosphate (dTMP). This protein equilibrates between an active and an inactive form, and between the dimer and the separated constituent monomers. The thymidylate cycle enzymes are important targets for anticancer drugs.^1^ Among the latter, methotrexate, raltitrexed (RTX), and pemetrexed (PMX) (Figure 1B) have been largely employed for a few decades. More recently, CT900 (ONX 0801) has been included in advanced clinical trials.^4^ At present, approximately 1800 ongoing clinical trials involve anticancer drugs that target hTS and other folate-dependent enzymes. All of these drugs are folate structural analogues that compete with the folate substrate to bind at the TS-enzyme active site. Their similarity to folic acid (FA) allows them not only to preferentially bind the folate enzymes but also to enter cells with the same mechanisms as FA, i.e., by folate receptor α (FRα), reduced folate carrier (RFC), and proton-coupled folate transport (PCFT).^5,6^ FRβ, an additional folate transporter, being mostly expressed in macrophages, is not relevant in the experimental model investigated in this work.

In the effort to discover new anticancer agents specifically targeting the TS cycle, we have recently identified some octapeptides, designed to target the protein monomer–monomer interface, that act as cell growth inhibitors of cisplatin (cDDP)-sensitive and -resistant human ovarian cancer (OC) cells.^7^ Among these, peptide LSCQLYQR (LR) and its isomer [DGln^4^]LR inhibit hTS activity.^8^ The X-ray crystal structures of the complexes with hTS of the LR peptide showed binding at the monomer–monomer interface of the inactive form of the enzyme (Figure 1C).^9−11^ Kinetic results were consistent with this unusual binding mode and with an inhibition mechanism based on stabilization of the inactive conformation of the enzyme. These peptides represent the only TS inhibitors that bind at the protein–protein interface, cause inhibition of cancer cell growth, and, at the same time, do not induce overexpression of hTS and lead to a reduced expression of the DHFR enzyme. This is at variance with the above-mentioned active site binding antifolate drugs that induce overexpression of both proteins, a fact likely related with the onset of drug resistance.^8^ The LR and [DGln^4^]LR peptides, however, feature poor cell membrane penetration and, to be delivered to cancer cells, require use of either a commercial peptide delivery system^8,9^ or untagged liposomes.^12,13^ While both delivery systems featured limited toxicity and gave interesting results, they did not allow a selective targeting of cancer cells. On the other hand, overexpression of specific transporters by some cancer types can provide an opportunity to develop a less laborious and more selective delivery approach.

A known cell-membrane-selective penetration strategy consists in combining FA with chemotherapeutic drugs yielding conjugates that are transported into cells by means of a physiological binder of FA. Once inside cells, the conjugates may either be cleaved to release the chemotherapeutic agent or, like in the case of CT900, act as such.^4^ Among the known folate transporters, PCFT only works at acidic pH values, while RFC has very low affinities for nonreduced FA.^6^ Thus, we have focused on folate receptor α (FRα). FRα is upregulated in many primary and metastatic cancers,^14^ including epithelial cancers and more than 90% of nonmucinous OCs,^15^ as well as in platinum-resistant ovarian cancers. Because it is almost absent in normal cells,^16^ this strategy is considered specific for cancer cells.^17,18^ As a result, targeted drug delivery via FRα promises to expand the therapeutic windows of drugs by favoring cancer cell membrane crossing and by increasing the drug distribution ratio between tumor and healthy tissues.^19−21^ Indeed, many clinical trials involving FA conjugates are ongoing.^22−25^

In this integrated experimental/computational work, we first describe the design and synthesis of the conjugates of FA with the two anticancer peptides, LR and [DGln^4^]LR (FA peptides). According to our design and molecular modeling description, the FA moiety is expected to allow the selective binding to FRα of an FA–peptide conjugate, with its ensuing endocytic internalization and release from the FRα:FA–peptide complex to interact with the TS target, as observed with other anticancer FA conjugates.^18−20^ Then, we tested the cytotoxic activities of the two conjugates versus several different cell lines characterized by different levels of FRα expression to show the FA–peptide conjugate preference for highly FRα-expressing cancer cells. The analysis of the modulation of a protein panel that was identified previously as a marker for the biological activity of the peptides suggests that the conjugates have the same intracellular mechanism of action as the free peptides. Finally, combination studies of the conjugate with anticancer agents were carried out to show the additive or synergistic effects in cancer cells with high and low FRα expression. These experiments confirmed the selectivity of compounds in the low concentration range of the conjugates.

In our design, we integrated molecular modeling analysis of the hTS:FA–peptide interaction complex and an experimental mechanistic investigation of the enzyme inhibition. Both analyses were fully consistent with these conjugates mainly acting as allosteric, dimer interface binding inhibitors. Overall, this multidisciplinary work represents an example of the design of lead compounds that addresses both cellular drug internalization and preservation of affinity toward the target enzyme. Also, it confirms that unconventional hTS inhibitors can bind at the enzyme monomer–monomer interface and exhibit anticancer activity without inducing overexpression of the target protein.

## Results and Discussion

### Design and Synthesis of the FA–Peptide Conjugates

We have designed the conjugates of folic acid, FA, with two peptides, LSCQLYQR (LR) and [DGln^4^]LR, with the aim to selectively internalize the FA–peptide conjugates in cancer cells through the FRα’s. Based on this design, we expect the FA moiety of each conjugate to bind FA binding site of an FRα, so promoting the endocytic cellular internalization of the conjugate. The FA moiety might also provide an additional hTS-binding mode for an FA–peptide conjugate. In fact, while, through its peptidic moiety, a conjugate can bind the enzyme at the monomer–monomer interface of the inactive form, the FA moiety might also directly bind at the folate binding site in the catalytic pocket of the enzyme.^2,7^

**Figure 2:**
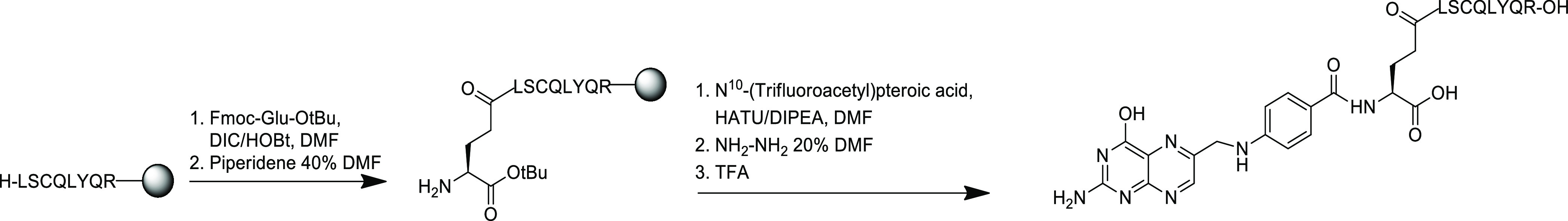
Synthesis of the FA–LR conjugate. A similar synthetic approach yielded FA–[DGln^4^]LR.

The FA–LR conjugate was synthesized according to the strategy outlined in Figure 2. Selective conjugation with a target molecule at the γ position of the glutamic moiety of FA is an essential requirement for recognition by FRα.^26,27^ However, our attempt to introduce a folate unit at the N-terminal position of LR as the last step of the solid-phase peptide synthesis (SPPS) of LR was unsuccessful. Because of the extremely low solubility of FA in all of the common solvents compatible with SPPS (i.e., dimethylformamide (DMF), *N*-methyl-2-pyrrolidone (NMP), dimethyl sulfoxide (DMSO)), and the difficulty to chemoselectively activate the γ carboxylic function of the glutamic moiety, we modified the synthetic strategy. The glutamic acid unit was chemoselectively introduced at the N-terminal of the LR peptide as the last step of the SPPS. Then, *N*^10^-(trifluoroacetyl)pteroic acid was condensed with [γGlu^0^]–LR, thus reassembling FA to give, after removal of the protecting groups, the final compound, FA–LR. The FA–[DGln^4^]LR conjugate was obtained following the same procedure. More details are given in the Supporting Information (SI) (Figure SI-1).

### Mechanism of Inhibition of Recombinant hTS

**Figure 3:**
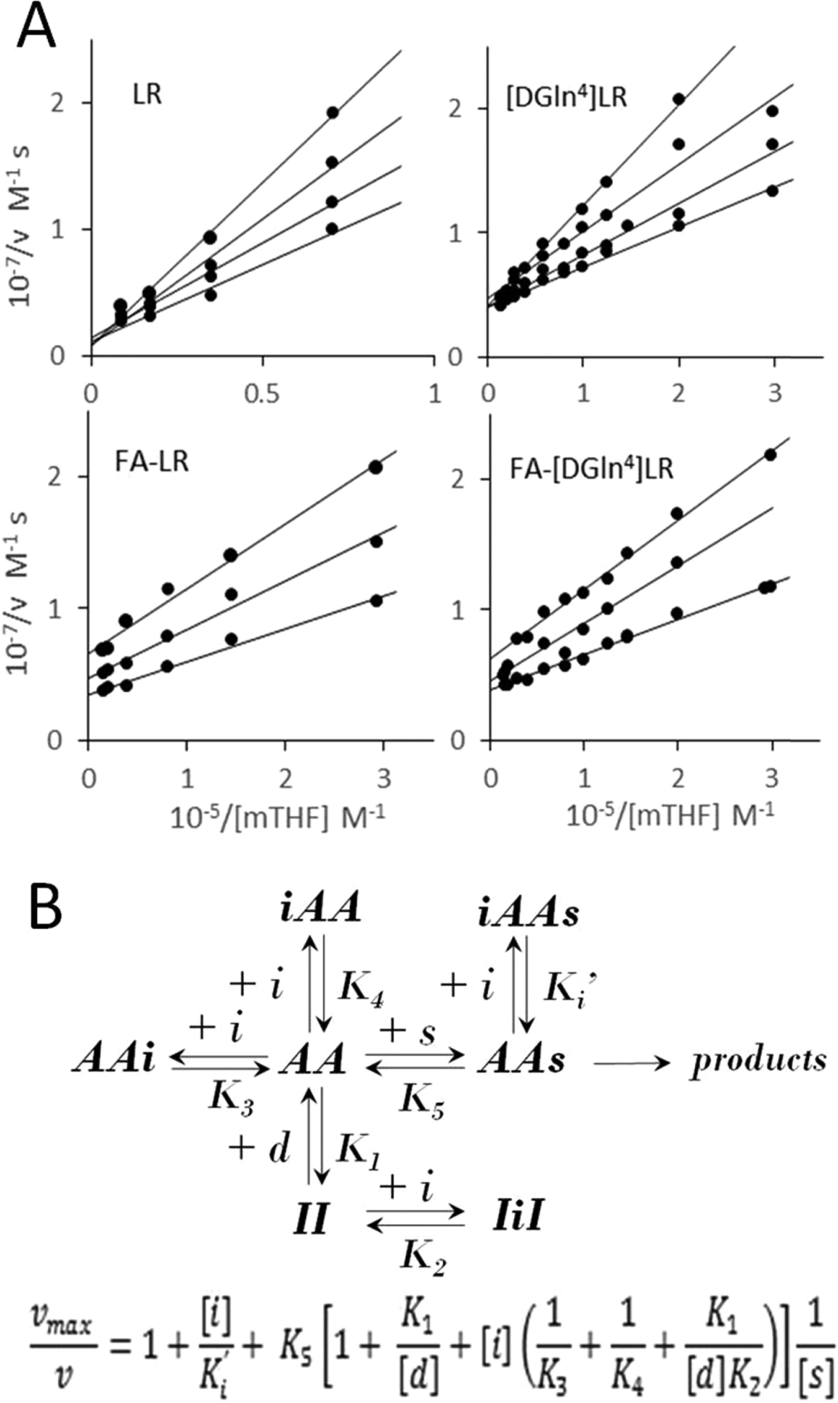
(A) Double-reciprocal plots for the inhibition of recombinant hTS (1 μM in the LR experiments, 350 nM otherwise) by peptides LR and [DGln^4^]LR and the bioconjugates FA–LR and FA–[DGln^4^]LR as a function of the mTHF substrate concentration. [LR] = 0, 25, 50, and 100 μM, [[DGln^4^]LR] = 0, 40, 80, and 150 μM, [FA–LR] = 0, 20, and 40 μM, [FA–[DGln^4^]LR] = 0, 50, and 75 μM. *v* = initial rate. [dUMP] = 140 μM. (B) Inhibition mechanism of hTS by the two peptides (only lower section, equilibria with *K*_1_ and *K*_2_) and the FA–peptide conjugates. II = inactive hTS dimer; AA = active hTS dimer–dUMP complex; d = dUMP; s = mTHF; i = inhibitor. Bottom: rate equation obtained by solving the mechanism under the fast-equilibration approximation.

We studied the inhibition of recombinant hTS by the two peptides, LR and [DGln^4^]LR, and their bioconjugates with FA at varying *N*^5^,*N*^10^-methylenetetrahydrofolate (mTHF) and inhibitor concentrations. The double-reciprocal plots in Figure 3A reveal two qualitatively different patterns of inhibition versus the folate substrate for the peptides and their FA conjugates. While the peptides exhibit formally competitive inhibition, the behaviors of the FA–peptide conjugates are better accounted for in terms of noncompetitive/mixed-type inhibition. The competitive-type inhibition, also exhibited by peptide LR versus the other substrate, 2′-deoxythymidine-5′-monophosphate (dTMP),^8^ is consistent with the inhibition mechanism sketched in Figure 3B. According to this mechanism, the two peptides bind the inactive form of the enzyme (I) at the monomer–monomer interface (species IiI and equilibrium 2 in Figure 3B). However, binding of mTHF (substrate s) to the active enzyme–dUMP complex (AA) shifts the coupled equilibria toward formation of the productive ternary complex (AAs), thus yielding a maximum initial rate independent of the peptidic inhibitor concentration, i.e., a formally competitive inhibition with common intercepts in the double-reciprocal plots. The slopes of the least-squares lines, fitted in terms of a linear dependence on the peptide concentration, yield apparent *K_i_* values of 90 ± 7 and 95 ± 13 μM for LR and [DGln^4^]LR, respectively. On the other hand, the double-reciprocal plots observed with the two FA–peptide conjugates indicate a dependence on the inhibitor concentration of both slopes and intercepts, i.e., of the apparent v_max_, thus a mixed-type inhibition. From the slopes, we obtain apparent *K_i_* values of 40 ± 15 and 73 ± 6 μM, and from the intercepts, we obtain apparent *K_i_*′ values of 44 ± 4 and 120 ± 30 μM for FA–LR and FA–[DGln^4^]LR, respectively. Overall, the latter is a slightly worse inhibitor than the former. In devising the inhibition mechanism in Figure 3B, that is an extension of the mechanism previously proposed for the competitive inhibition by the LR peptide,^8^ we have taken into account the difunctional nature of the FA–peptide inhibitors and have assumed formation of significant amounts of nonproductive or slowly productive enzyme–substrate–inhibitor complexes (iAAs). Similarly to our findings, three folate analogues and their polyglutamylated forms were found to act as noncompetitive or mixed inhibitors depending on their relative affinities for the folate binding sites in the two catalytic pockets of hTS, the latter claimed to become asymmetric and feature strongly different affinities for the folate substrate as a result of dUMP binding.^28^ From this study, we borrow the idea that folate substrate analogues can bind hTS at two different sites. They can both compete with the mTHF substrate at the catalytic pocket of the monomer occupied by dUMP (AAi, equilibrium 3 in Figure 3B) and bind at the catalytic pocket of the other monomer (iAA), possibly yielding a nonproductive complex together with the folate substrate (iAAs, equilibria 4 and 6). According to the kinetic equation obtained by solving the mechanism in Figure 3B, and the assumption that the main inhibition route of the peptides is the formation of the IiI complex, for the two peptides, the apparent *K_i_*’s correspond to [d]*K*_2_/*K*_1_, where d is dUMP. As in our experiments [d]/*K*_1_ holds about 15, from the two apparent *K_i_*’s, 90 and 95 μM for LR and [DGln^4^]LR, respectively, we estimate *K*_2_ values 6–7 μM. These figures are consistent both with the value estimated for the same equilibrium constant from inhibition experiments performed with LR at fixed mTHF (∼10 μM)^8^ and with the fluorometrically evaluated dissociation constant of the complex of the LR peptide with hTS in a cellular environment (*K*_d_ = 3.4 ± 0.5 μM).^29^ Because 1/*K_i_* = 1/*K*_3_ + 1/*K*_4_ + *K*_1_/[d]*K*_2_, the lower apparent *K_i_* values of the FA–octapeptide conjugates, 38 and 73 μM, with respect to the *K_i_*’s of the peptides, lend support to the idea that the former can exert two kinds of competitive-type inhibition, i.e., binding of the peptidic moiety at the monomer–monomer interface of the inactive form of the enzyme (IiI, equilibrium 2 in Figure 3B) and of the FA-end to the hTS–dUMP binary complex at the catalytic site(s) (iAA and AAi, equilibria 3 and 4). If we assume the *K*_2_ values to be similar for the conjugates and peptides, we estimate the 1/*K*_3_ + 1/*K*_4_ term to hold 0.015 and 0.003 μM^–1^ and calculate the harmonic means of *K*_3_ and *K*_4_ as 130 and 630 μM for FA–LR and FA–[DGln^4^]LR, respectively. From these values, we argue that the former conjugate is more efficient than the second one in the additional path of competitive inhibition that, according to our kinetic model, corresponds to binding of the FA-end of the conjugates at the active site(s) (equilibria 3 and 4). The higher propensity of FA–LR for this binding is confirmed by the apparent *K_i_*′ values, 44 versus 110 μM. In fact, according to our kinetic model, this constant corresponds to *K*_4_*K*_6_/*K*_5_ and is then closely related to the *K*_4_ constant. However, the affinity of this binding at the active site of the enzyme is at least 1 order of magnitude lower than that at the monomer–monomer interface for FA–LR and even lower for FA–[DGln^4^]LR. The 2-fold binding potential of the FA–peptide conjugates and the higher efficiency of the noncompetitive inhibitory behavior of FA–LR with respect to FA–[DGln^4^]LR are consistent with the molecular modeling results described below.

### Molecular Modeling of the Interaction of FA–LR and FA–[DGln^4^]LR with hTS

**Figure 4:**
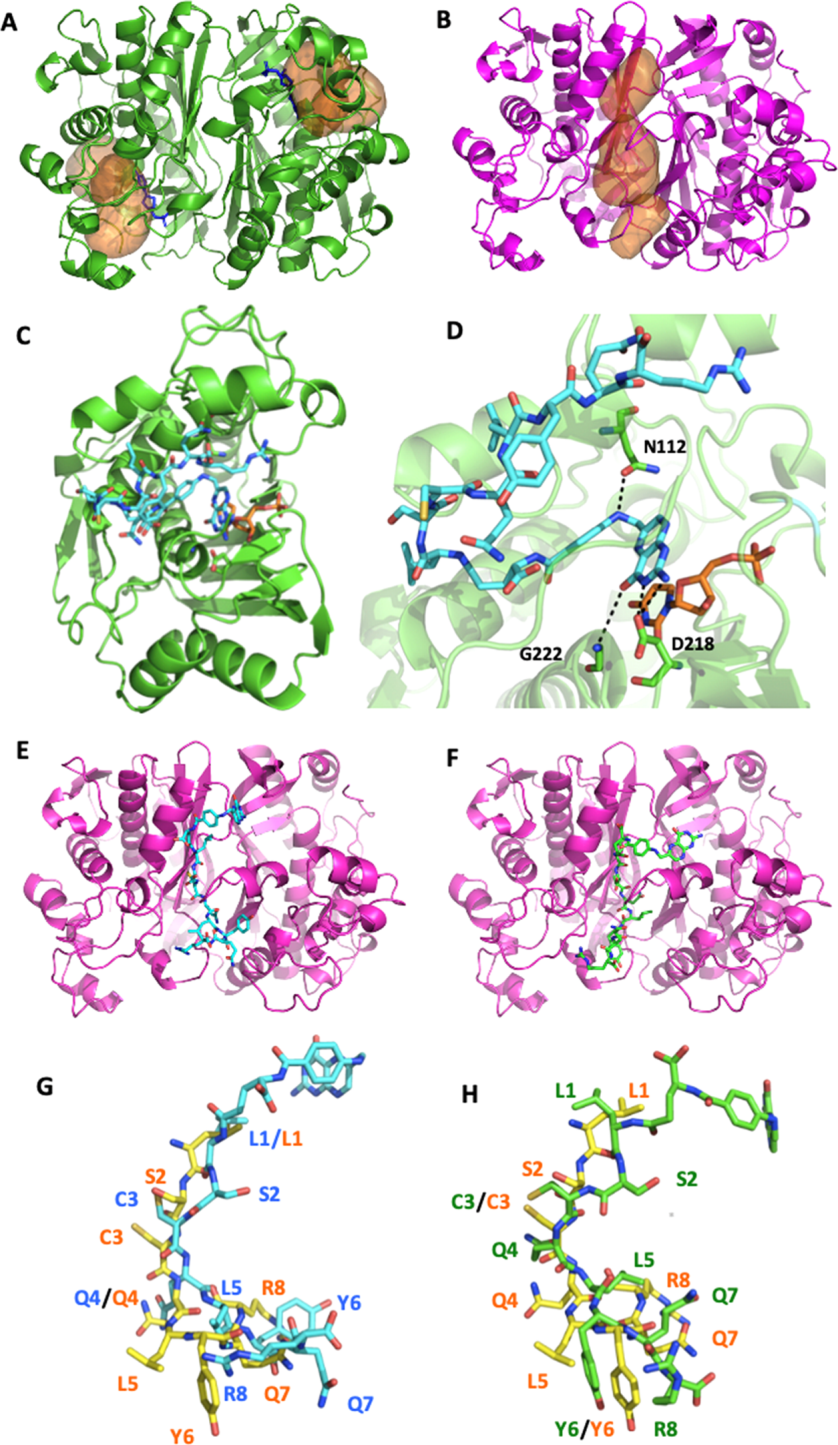
hTS structure and docking simulation of FA–LR and FA–[DGln^4^]LR in the active and inactive protein conformations. (A) hTS active form (PDB code 1HVY). The two binding sites are represented by orange surfaces, the dUMP substrate in blue sticks. (B) hTS inactive form. The large cavity at the protein subunit interface is represented by orange surfaces. (C) Docking pose of FA–LR within the hTS active form. For clarity, only one monomer has been reported. The ligand and the dUMP substrate are shown in cyan and orange capped sticks, respectively. (D) Close-up of the FA–LR/hTS docking complex in the active site. Hydrogen bonds are shown as black dashed lines. Protein residues are labeled and displayed as green capped sticks. (E) Docking pose of FA–LR at the protein subunit interface of the hTS inactive form (IiI state in Figure 3B). (F) Docking pose of FA–[DGln^4^]LR at the protein subunit interface. (G, H) Superimposition of the docking pose of FA–LR (7, cyan capped sticks) and FA–[DGln^4^]LR (8, green capped sticks) at the protein subunit interface with the structure of the LR peptide co-crystallized with inactive hTS (yellow capped sticks, PDB code 3N5E). The labels indicate the position of the residue side chain (light blue, FA–LR; green, FA–[DGln^4^]LR; orange, LR).

Human TS can assume both an active and an inactive conformation (Figure 4).^30,31^ In the di-active form (Figure 4A), the two catalytic sites, one per monomer, are occupied by the dUMP substrate. FA and folate analogues are known to bind the dUMP-bound active form and to interact with the dUMP substrate mostly by π–π interactions.^31^ In the inactive form, found only in the absence of the dUMP substrate (Figure 4B), the binding sites enlarge and a broad cavity appears at the monomer–monomer interface, where the LR peptide can bind (IiI state in Figures 3B and S1-3).^8^ Therefore, the active/inactive hTS conformations represent relevant models for studying the interaction with FA–peptide conjugates. The latter can exploit this binding opportunity through their peptidic moieties and can still bind the catalytic site of the active form by means of their folate moiety, in the presence of dUMP (iAA, AAi, and iAAs in Figure 3B). To gain insight into these conjugate hTS-binding modes, we performed molecular docking simulations in both the di-active and di-inactive protein conformations. Docking of FA–LR into the active site of the hTS active form (PDB code 1HVY)^32^ led to the formation of the complex reported in Figure 4C,D. The docking region is represented by the pocket including the dUMP substrate and by a part of the outer solvent-exposed region. The FA–LR folate moiety assumes an orientation similar to those of FA and antifolate inhibitors, interacting with Asn112 through the aminobenzoate nitrogen (Figure 4D), but also with Asp218 and Gly222.

The ability of the folate moiety to properly occupy the folate region can be appreciated by superimposing the FA–LR docked pose with the crystallographic orientation of raltitrexed (Figure SI-2).^32^ The peptidic chain of FA–LR hangs out of the binding pocket and takes a folded orientation making transient contact with Ser114 and Phe117, through the side chain of the glutamine at position 7. Notably, only 2 docking runs, over 25, generated the mentioned conformation and properly located the folate moiety within the binding site, thus underling the difficulty of the conjugate to adjust within the orthosteric site of the enzyme active form. Despite the difficulty of docking FA–LR in the active site, the conformation reported in Figure 4C,D obtained a good GoldScore of 85. When raltitrexed was re-docked in the co-crystallized structure (PDB ID 1HVYHVY), all poses were homogeneous and able to reproduce the crystallographic conformation (Figure SI.2). The best pose was scored 55, so lower with respect to that of FA–LR, possibly because of the additional number of interactions made by the LR chain. Docking FA–[DGln^4^]LR in the hTS active site gave worse results, as no feasible pose into the folate pocket was obtained, possibly because of the higher rigidity of the conjugate peptidic region. Overall, this suggests a significant difficulty for the two conjugates, particularly for FA–[DGln^4^]LR, to enter the folate binding site in the active form of the protein. We did not attempt any binding at the protein interface because in the active conformation there is no room for hosting compounds at this interface. Both conjugates were then docked at the protein subunit interface of the inactive hTS conformation using the crevice at such interface as a possible binding site (PDB code 3N5E).^32^ The docking poses for FA–LR and FA–[DGln^4^]LR are reported in Figure 4E,F, respectively. In both complexes, the central peptidic region of the ligand lies at the monomer–monomer interface, while the folate moiety and the peptide tail are oriented toward a more solvent-exposed region. Moreover, the peptide orientation may allow the formation of a disulfide bond between the cysteine in position 3 of the peptide and cysteine 180 of hTS. According to the crystallographic structure of the hTS–LR complex, in fact, the cysteine sulfhydryl group lies at a suitable distance to form an S–S bond with the peptide.^8^ The superimposition of the docking poses of FA–LR and FA–[DGln^4^]LR with the structure of the LR peptide co-crystallized at the interface of hTS inactive form (Figure 4G,H) highlights the similarity between the predicted (docking) and the experimental (crystallographic) orientation of the peptidic region. A detailed description of the conjugates pose at the enzyme interface is reported in the SI. Briefly, FA–LR H-bonds to Gly143 in both chains, Arg163, Thr170, and Leu192 in chain B (Figure SI-3a), while FA–[DGln^4^]LR H-bonds Ala166, Pro169 in chain A Ala119, Thr142, and Thr145 in chain B (Figure SI-3b). Overall, the docking simulations confirmed the capability of both conjugates to easily bind the protein interface without the need to impose any structural constraint, in keeping with the previously described competitive inhibition ability of these compounds.^8^ In particular, when docked at the interface of hTS inactive form, the FA–LR and FA–[DGln^4^]LR conjugates were scored 79 and 76, respectively, quite similarly to the LR conjugate when re-docked in the corresponding X-ray structure (PDB ID 3N5E, GoldScore equal to 84).

As previously mentioned, docking the conjugates at the binding site in the hTS active conformation in the presence of dUMP turned out to be more difficult in the case of FA–LR and not possible with FA–[DGln^4^]LR. These findings support the indications from the kinetic analysis that both conjugates bind more easily at the dimer interface than at the protein active site. This preference might shift the active–inactive equilibrium toward the inactive state of the enzyme, in the presence of the conjugates.

These findings support the indications from the kinetic analysis that both conjugates bind more easily at the dimer interface than at the protein active site and, concerning a comparison between FA–LR and its more rigid DGln^4^ analogue, are consistent with the experimental observation of smaller values of *K_i_*′ and, particularly, of the harmonic mean of *K*_3_ and *K*_4_ for the former conjugate with respect to the latter.

### Expression of FRα by Ovarian Cancer Cell Models

**Figure 5:**
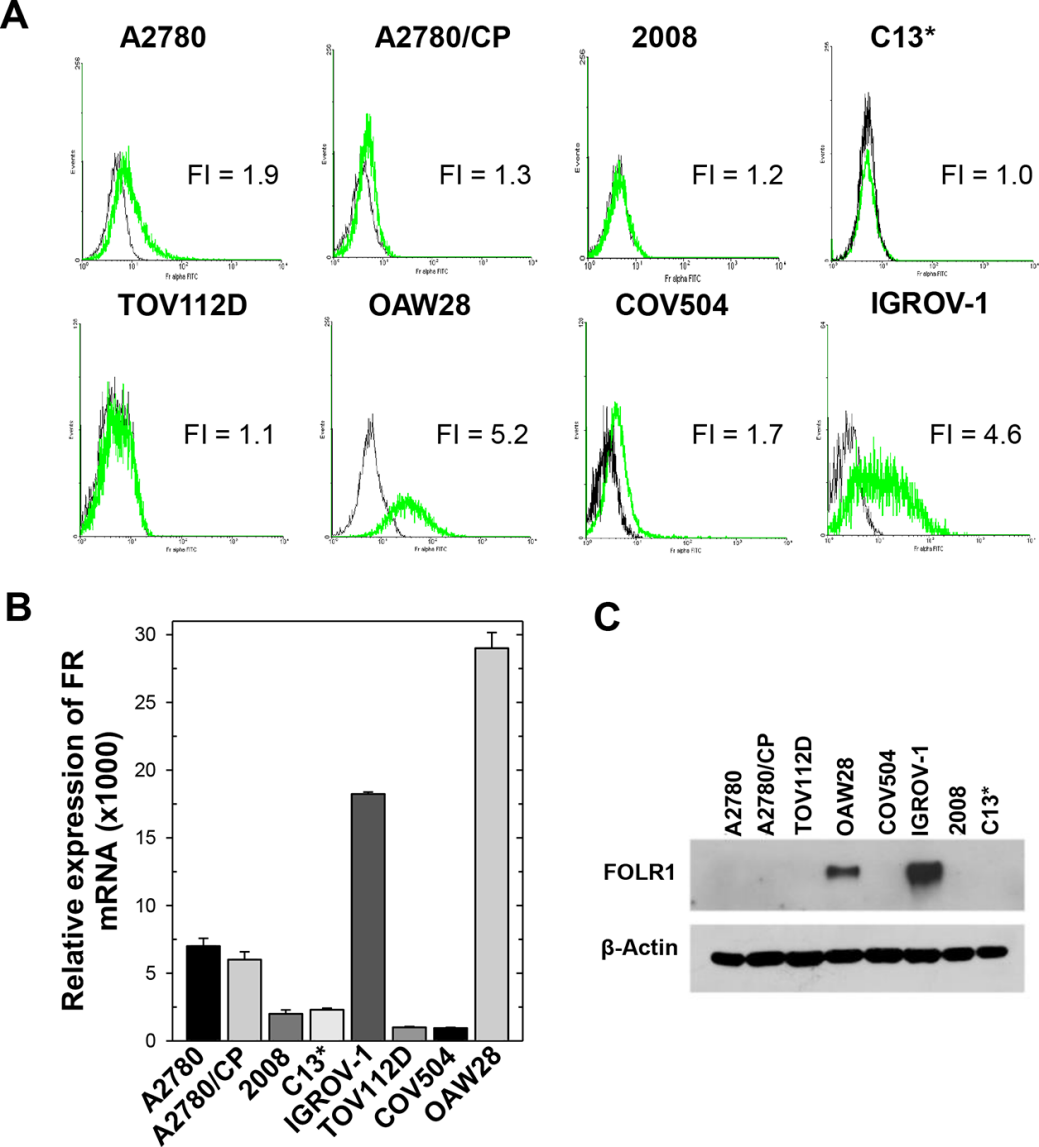
Expression of FRα in a panel of eight cancer cell lines. (A) Flow cytometric assessment of FRα expression on cell surface; black: cells labeled with secondary antibody alone without anti-FRα Mov18 antibody; green: cells labeled with anti-FRα Mov18 antibody; FI: ratio of the mean fluorescence intensities in the presence and absence of the primary antibody. (B) Quantitative PCR (qPCR) measurements of FRα transcript in the eight cell lines; *y* axis: levels of FRα mRNA relative to GAPDH mRNA. (C) Western blot reporting FRα protein (FOLR1) at 38 kDa in the cell line extracts reported in the horizontal bar. Representative blots of three independent experiments are shown. Human β-actin was used as internal control for protein loading. Each data point represents the mean ± standard error of the mean (S.E.M.) of three separate determinations.

To test the preference of the FA–peptide conjugates for highly FRα-expressing cell lines and evaluate the efficiency of the corresponding mechanism of transport through the cell membrane and the ability of the internalized conjugates to induce cell growth inhibition, we have selected a panel of eight ovarian cancer cell models. Since the efficiency of cellular internalization of drugs by FRα depends on the expression of this surface protein, we first investigated its levels in some OC cell lines. The OAW28, COV504, IGROV1, TOV112D, 2008, C13*, A2780, and A2780/CP cell lines, previously histologically and morphologically well characterized, were selected for their histological differences as well as for the sensitivity to cisplatin.^33,34^ C13* and A2780/CP are cisplatin-resistant cells that feature high hTS expression levels resulting from a resistance mechanism induced by cisplatin treatment. On the other hand, 2008 and A2780 are cisplatin-sensitive cells and feature intermediate hTS protein levels.^35,36^ We performed this analysis using both quantitative and semiquantitative methods, including flow cytometry, Western blot (WB), quantitative real-time polymerase chain reaction (PCR) and radioligand binding assays.^37−39^ Flow cytometry results indicated that the OAW28 and IGROV-1 cells expressed the largest amounts of the FRα receptor (Figure 5A). Again, high levels of FRα-mRNA (relative mRNA expression, *y* > 10 in Figure 5B) characterized the IGROV1 and OAW28 cell lines, intermediate levels the A2780 and A2780/CP cells (5 < *y* < 10), and very low levels the other four cell lines, particularly the TOV112D and COV504 cells (*y* < 5, Figure 5B). Consistently, in the WB analysis, bands due to total FRα were detected only with the OAW28 and IGROV1 cells, the latter showing the highest FRα cellular amount (Figure 5C). To evaluate the level of functional FRα, a crucial property for FR-targeted therapies,^40^ we performed radioligand binding assays. The IGROV1 cells exhibited the highest amount of functional FRα on their surface, more than 90 fmol of [^3^H]FA bound per mg of protein versus about 40 fmol/mg for OAW28 cells and values lower than 30 fmol/mg for the other cell lines (Figure SI-4 and Table SI-1).

### FA–LR Binding to FRα and Cellular Uptake

**Figure 6:**
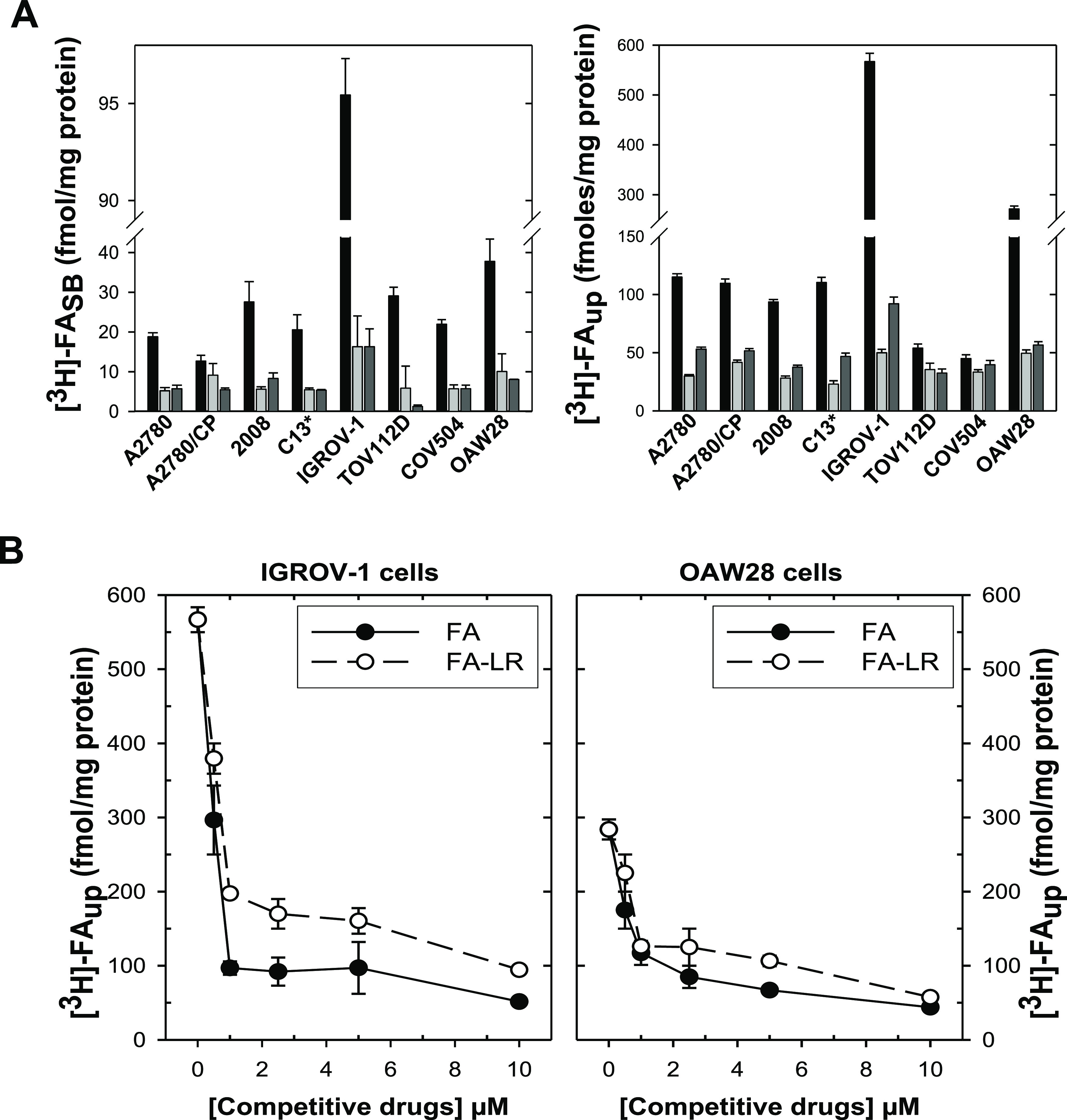
(A) Femtomoles per mg of protein of [^3^H]FA bound to the cell surface (left, [^3^H]FASB) and uptaken (right, [^3^H]FAUP) by human ovarian cancer cell lines. Column color code: black, [FA] = [FA–LR] = 0; pale gray, [FA–LR] = 0, [FA] = 5 μM; dark gray, [FA–LR] = 5 μM. (B) Dependence of [^3^H]FAup on the concentrations of FA (black circles) and of FA–LR (white circles) in IGROV-1 (left) and OWA28 cells (right). Each data point represents the mean ± S.E.M. of three separate determinations. SB = surface binding; up = uptake.

We then investigated the affinities for FRα of FA and the FA–LR bioconjugate by measuring their abilities to compete with [^3^H]FA for binding FRα on the cell surfaces. Binding of [^3^H]FA to the FRα of all cell lines was greatly and similarly inhibited by unlabeled FA and the FA–LR conjugate (Figure 6A, left). The inhibition was more pronounced with highly FRα-expressing cells, i.e., IGROV1 and OAW28 (−80 and −70%, respectively, of bound [^3^H]FA at 5 μM FA–LR). As an expected consequence of the competitive binding to FRα, [^3^H]FA uptake was reduced in the eight cell lines by both unlabeled FA^39^ and the FA–LR conjugate (Figure 6A, right). We found that while in most cell lines competition with FA–LR caused a decrease of [^3^H]FA uptake by 50% or less, this decrease reached about 80% (from 583.3 ± 42 to 92.2 ± 11 fmol/mg of protein) and 75% (from 271.5 ± 27 to 56.6 ± 8 fmol/mg of protein) with IGROV1 and OAW28 cells, respectively, i.e., with highly FRα-expressing cells.

Then, we used these two cell lines to investigate quantitatively the ability of FA–LR to directly compete with folic acid for binding to cell surface FRα. The corresponding dose–response plots in Figure 6B indicate that [^3^H]FA uptake by both cell lines is efficiently inhibited by FA–LR and unlabeled FA already at about 500 nM, and decreases more slowly, or remains almost constant, at concentrations up to 2 μM. This rapidly saturating trend is likely connected with engagement of all cellular FRα by either of the two [^3^H]FA competitors already at 500 nM. It adds to the observation of higher competitive effects on [^3^H]FA uptake on cancer cells (IGROV1 and OAW28) that express larger amounts of FRα in supporting the crucial role of these receptors in the cellular internalization of the FA–LR conjugate.

Given the structural similarity between FA–LR and FA–[DGln^4^]LR, we have limited these competitive-uptake experiments to the former. Indeed, the computational investigation of the interaction with FRα reported in the following paragraph strongly suggests that the binding mode to FRα is the same for the peptidic moieties of the two conjugates.

### Binding Modes of FA–LR and FA–[DGln^4^]LR to FRα

**Figure 7:**
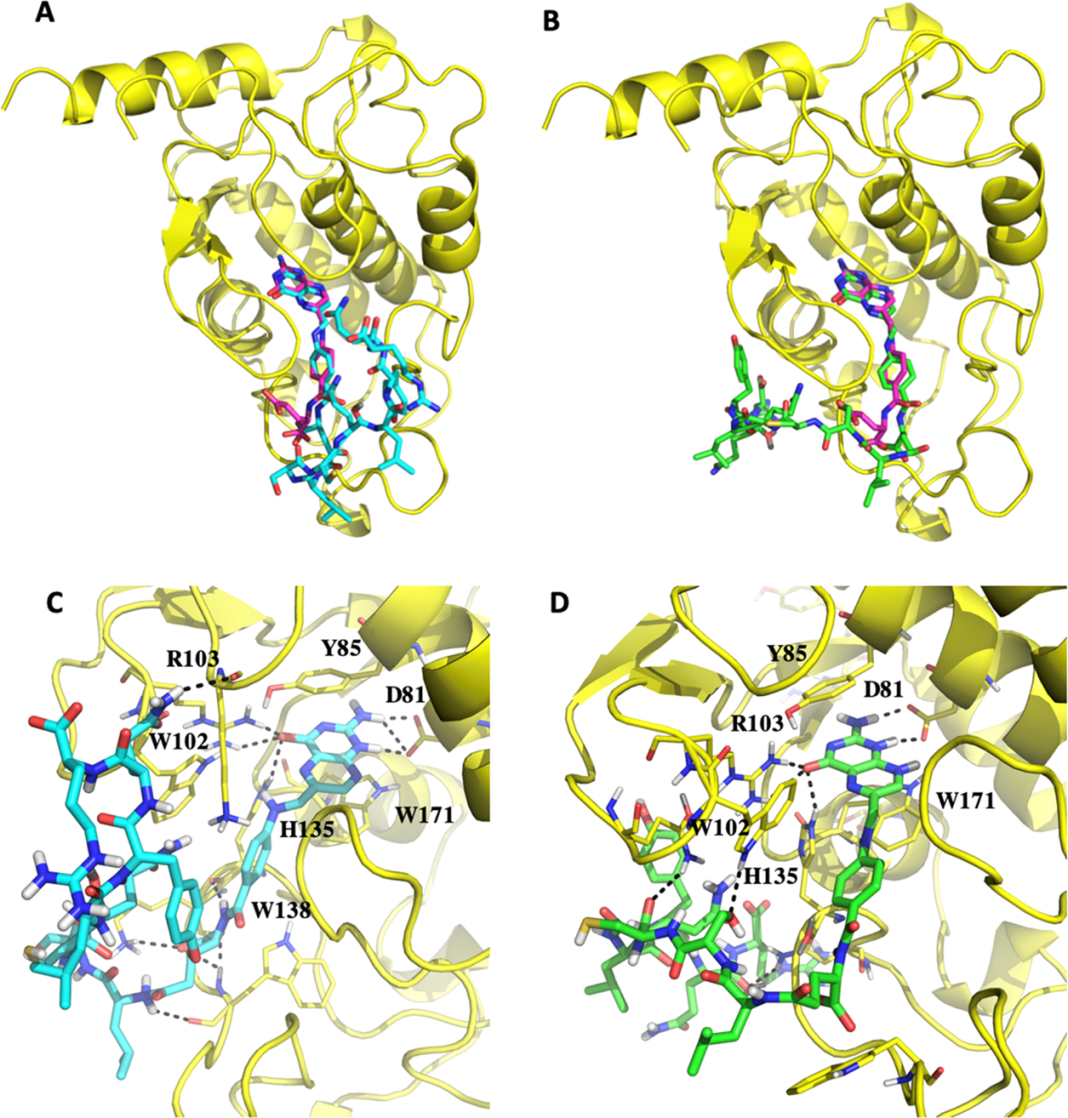
Docking pose of the FA–LR ((A) cyan capped sticks) and FA–[DGln^4^]LR ((B) green capped sticks) conjugates in FRα. The co-crystallized FA is displayed for comparison in magenta sticks (PDB code 4LRH). The protein is represented in cartoons. (C, D) Insight of the FA–LR/FRα and FA–[DGln^4^]LR/FRα complexes. Residues lining the pocket and interacting with the conjugates are shown in capped sticks. Crucial residues are labeled. Images were prepared with Pymol version 1.7.0.0.

To test the ability of the FA–LR and FA–[DGln^4^]LR conjugates to bind FRα, we have carried out a comparative investigation of these interactions by docking simulations using the GOLD software (Figure 7). The crystallographic structure of FAα complexed with FA^41^ (PDB code 4LRH) was used as a template after ligand removal. In the X-ray structure, FA is strongly bound to the receptor by means of several interactions. The pteridine moiety forms H-bonds with Asp81, Arg103, Arg106, His135, and Ser174, and π–π interactions with Tyr85 and Trp171. The phenyl ring also forms a π–π interaction with Trp102, while the amidic nitrogen contacts His135. Finally, the two carboxylic groups interact with Gln100, Trp102, Gly137, Trp138, and Trp140.^42^ When docked in the FAα folate binding site, the FA–LR conjugate assumes a plausible orientation, resembling that of FA (Figure 7A). The hydrogen bonds with Asp81, Arg103, and His135 are conserved (Figure 7C). The amidic nitrogen contacts the backbone carbonyl of His135, while the carboxylic moiety interacts with the backbone amino groups of Lys136 and Trp138. π–π interactions are made by the pteridine group with the aromatic systems of Tyr85 and Trp171, and by the benzoate group with Trp102. Additional contacts are formed by the peptidic tail with other residues facing the binding pocket entrance or localized on the receptor surface. In particular, H-bonds are formed with the backbone of Lys19 and Trp138. Overall, the FA–LR conformation reported in Figure 6A was scored 93, while the redocked FA obtained a GoldScore equal to 73.

These findings support the capability of the FA–LR conjugate to bind to the cell surface FAα receptor and compete with FA, as shown in the previous paragraph. Similar interactions were found when looking at the docking pose of the FA–[DGln^4^]LR conjugate in the same FRα binding site (Figure 7B,D). In particular, the pteridine ring takes the same orientation and maintains a similar number of H-bonds and π–π interactions, while the peptidic tail takes a different orientation stabilized by H-bonds with Gln100, Trp102, and Asn133 side chains. This conformation obtained a GoldScore of 126. It should be mentioned that in the different poses obtained by the docking simulations, the position of the tail was not conserved, and that different conformations stabilized by diverse contact were generated. This is quite reasonable, given the length of the chain and the rather globular surface of the protein. From the computational perspective, we find no significant differences between the binding modes of the FA fragments of the two bioconjugates; thus, formation of the two protein–conjugate complexes seems to be equally favorable.

### Compartmentalization of FA–LR in Cancer Cells

The amounts of FA–LR in the extracellular medium and in the vesicles (endosomes) and the cytosol of IGROV-1 cells were measured by liquid chromatography with tandem mass spectrometry (LC-MS/MS) (Agilent 6410) following a 30 min cell incubation with 5 μM conjugate (Table 1). The experiments were performed both at 37 °C, with the FRα’s fully active, and at 4 °C, with the FRα-mediated endocytic process drastically reduced. The FA–LR content measured in the cytosol and vesicles was converted into cytosolic and intravesicular molar concentrations using a mean cellular volume of 2 × 10^–12^ L,^43^ mean cytosolic and vesicular volumes, respectively, 60 and 2.5% of the cellular volume and a total number of 2 × 10^6^ lysed cells. Further details of the development and of an analytical validation of this method are provided in the Experimental Procedures section and in the Supporting Information (Tables SI-2–SI-7 and Figure SI-5).

**Table 1:** Mass (ng = Nanograms) and Molar Concentration of the FA–LR Conjugate in the Extracellular Medium, in the Cytosol, and in Vesicles Following a 30 min Incubation of IGROV-1 Cells with 5 μM FA–LR (Mean Value ± Standard Deviation, *n* = 6)

	*T* = 37 °C		*T* = 4 °C	
fraction	FA–LR mass (ng)	[FA–LR] (μM)	FA–LR mass (ng)	[FA–LR] (μM)
extracellular	10 200 ± 1200	2.38 ± 0.28	19 300 ± 2200	4.48 ± 0.50
cytosolic	60.2 ± 8.6	18.5 ± 2.6	31.6 ± 5.7	9.7 ± 1.8
vesicles	34.4 ± 4.3	254	18.6 ± 2.9	137

The cytosolic concentration found with the 37 °C incubation (18.5 ± 2.6 μM) was confirmed by a second experiment based on fluorescence, a completely independent observable. The pteroate moiety of FA exhibits a characteristic fluorescence emission^44^ that can be enhanced by UV-light-induced oxidative photocleavage of the bond between the fluorophore and the *p*-aminobenzoate unit (the latter acts as a quencher).^45^ Emission and excitation measurements on IGROV-1 cell lysates provided pteroate signals that increased upon repeated exposures to the excitation lamp up to final, stable values (Figure SI-6). The experiments yielded average emission intensities at 445 nm of 834 ± 32 kcounts from the cells treated with a 5 μM FA–LR conjugate solution and 747 ± 36 kcounts from the untreated cells; the average excitation signals at 360 nm were 1120 ± 25 and 994 ± 80 kcounts for the treated and untreated samples, respectively. The differences between positive and control signals, proportional to the uptaken FA–LR concentrations, were therefore 87 ± 48 and 126 ± 84 kcounts, respectively. Using a previously determined calibration factor, the number of cells (around 6 × 10^6^), an average cell volume of 2 × 10^–12^ L,^43^ and a typical cytosolic volume of 60% the cellular volume, we estimate the corresponding cytosolic concentrations to be 20.2 ± 11 and 24.3 ± 16.1 μM from FA–LR emission and excitation signals, respectively. Thus, under these conditions, the fluorometric analysis confirms the LC-MS result of a 4-fold increase of FA–LR concentration within IGROV-1 cells relative to the external incubation medium.

Given the relevance of the concentration data, we checked whether the 30 min incubation time could represent a drawback in our analytical approach. In the extracellular matrix, the conjugates were stable in the first 30 min, at 4 °C and 37 °C (data not shown). The evolution with time of the cytoplasmic concentration of the FA–LR peptide in C13* cells treated at 37 °C with a medium containing 5 μM FA–LR was analyzed using LC-MS Orbitrap Q-Exactive. We determined the concentration of FA–LR after 20 min from the administration of the compound to the cancer cells, and then after 40, 60 min, and then 2 and 4 h (Figures SI-7 and SI-8). The concentration decreased with a first-order kinetics, the rate constant and half-life being respectively 3.5 (±0.5) × 10^–3^ min^–1^ and 200 (±30) min (Figure SI-8). Therefore, because the cytosolic FA–LR conjugate is degraded in a time scale 1 order of magnitude longer than the timing of our analytical experiments, we can assume our results to characterize a steady-state, if not an equilibrium, situation.

The FA–LR concentration ratio found between the vesicular and the cytosolic fractions (13.7 at 37 °C, Table 1) is similar to the ratio of concentrations (10.8) of tritiated FA reported in the same two cellular fractions of R1-11-FR2 and R5-FR12 HeLa cells separated with the same procedure used by us.^39^ This similarity lends support to the reliability of the analytical tools used in this work and confirms the crucial role played by the folate moiety of the FA–LR conjugate in its ability to cross the cell membrane.

Among the possible internalization pathways of the FA–peptide conjugates that exploit the FA moiety, the process mediated by the reduced folate carrier (RFC) is likely negligible in our experiments. In fact, under physiological conditions, FA features a *K*_m_ > 100 μM toward RFC,^46^ and at [FA–LR] = 5 μM, the fraction of RFC bound to the conjugate is very low. As for PCFT, this proton symporter contributes to the cellular uptake of folates and antifolates at acidic pH, usually fixed at 5.5 in published reports.^5,47^ Therefore, its contribution is likely negligible at pH values 7.2 and 7.4, at which our uptake experiments were carried out. To further support this statement, we performed a few experiments on 2008, C13, A2780 and A2780/CP cells at pH 8 and found no significant change in the [^3^H]FA uptake results.^48,49^

As for the FRα-mediated endocytosis, the amounts and concentrations measured at 4 °C, i.e., conditions under which it is believed to be little efficient, were approximately half those determined at 37 °C, when it is fully active (Table 1). This, together with the 14-fold larger concentration of FA–LR in vesicles than in the cytosol, points to a relevant role for the endocytosis-mediated internalization process. Such a relevance is further supported by the cytotoxicity results in the following paragraph.

On the basis of the available figures, we expect that selectivity for the FRα-mediated cellular internalization path be enhanced at lower conjugate concentrations, e.g., at hundreds of nanomolar concentrations (100–500 nM) that are likely sufficient to saturate these cell surface receptors (Figure 6B).

### In Vitro Activity of the FA–LR and FA–[DGLN^4^]LR Conjugates

**Figure 8:**
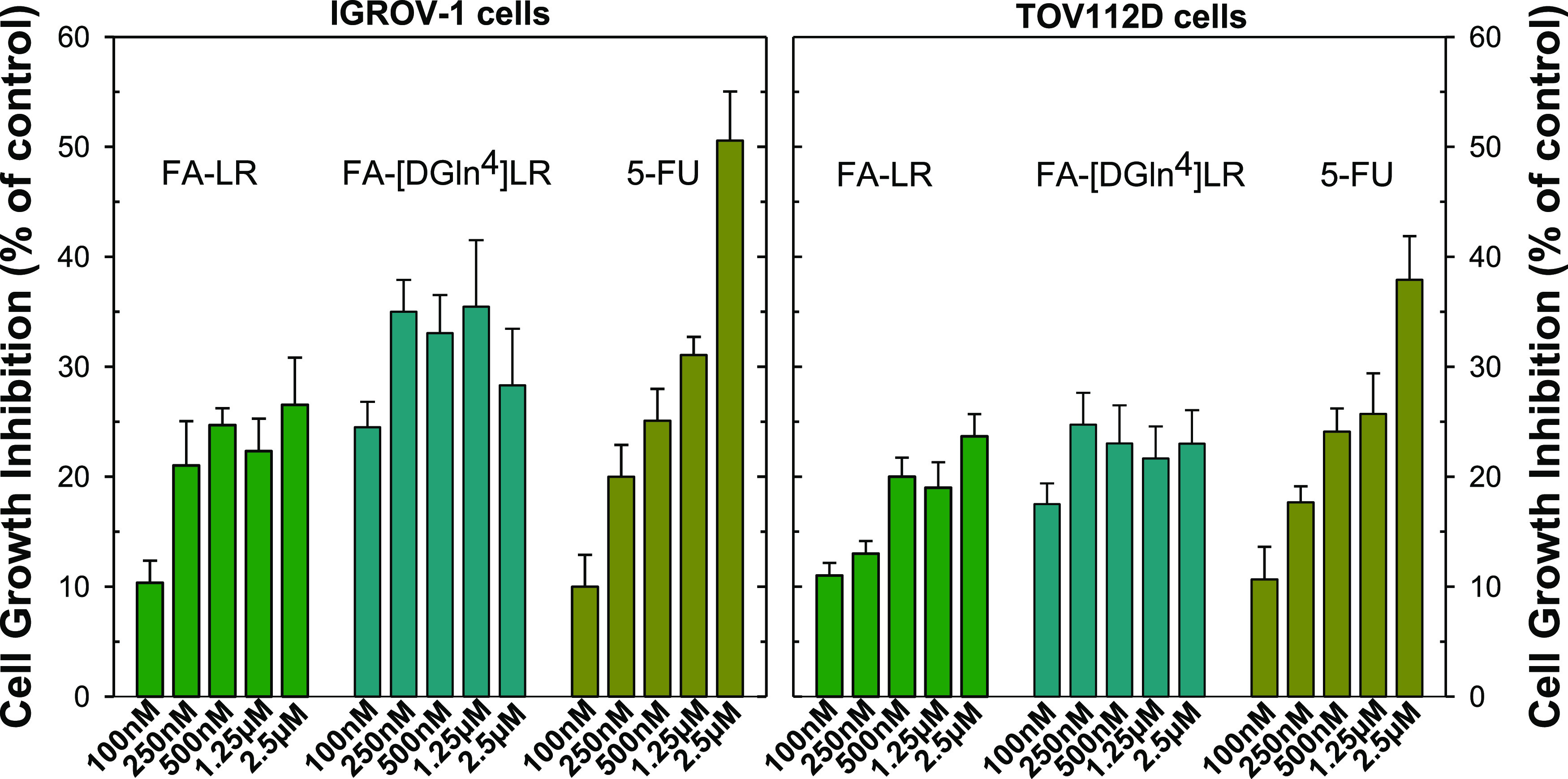
Growth inhibition of IGROV-1 and TOV112D cells by FA–LR, FA–[DGln^4^]LR, and 5-FU. And 24 h after seeding in complete medium, this was replaced with folate-free (FF) medium and the cells were treated with increasing concentrations of the bioconjugates and 5-FU three times every 12 h. Then, the cells were allowed to grow up to 72 h. All of the results plotted represent the mean of three separate experiments performed in duplicate. Error bars, S.E.M.

We have previously reported the cytotoxic activities of the LR and [DGln^4^] LR peptides transfected into cells by means of either the specific peptide delivery system, SAINT-PhD,^8^ or liposomes.^12,13^ We now compare them with the cytotoxic effects of the FA–peptide conjugates administered as such at concentrations ranging from 100 nM to a few micromolar (Figure 8). The compounds were tested against the six cancer cell lines in Figure 6 (IGROV1, OWA28, 2008, C13*, A2780, and A2780/CP). Among them, the IGROV1 and OWA28 lines, i.e., those that express the highest levels of FRα, showed a growth inhibition effect after treatment with the FA–peptide conjugates. Of the lines that express low levels of FRα, the 2008, C13*, A2780, and A2780/CP cells did not respond to direct treatment with the conjugate and showed a response only after treatment with the conjugates delivered with the SAINT-PhD delivery system (Figure SI-9). Only the TOV112D cells showed a cytotoxic response and were chosen as a little FRα-expressing reference cell line.

Already in the 100–500 nM concentration range, both conjugates caused an IGROV1 cell growth inhibition of about 20–35%, though in a weakly dose-dependent manner (Figure 8, left). The FA–[DGln^4^]LR derivative displayed a slightly higher efficacy than FA–LR, somewhat reminiscent of the larger cytotoxicity of [DGln^4^]LR with respect to LR when administered with the SAINT-PhD peptide delivery system.^8,9^ At 100 nM for both conjugates, and up to 500 nM for FA–[DGln^4^]LR, the cell growth inhibition was even larger than that of the reference drug, 5-FU (Figure 8). The FA–[DGln^4^]LR conjugate showed a slightly yet consistently higher activity toward IGROV-1 than toward TOV112D cells (Figure 8, right), a finding likely related with the higher expression of FRα in the former cells (see Figure 5 and the corresponding paragraph).

**Figure 9:**
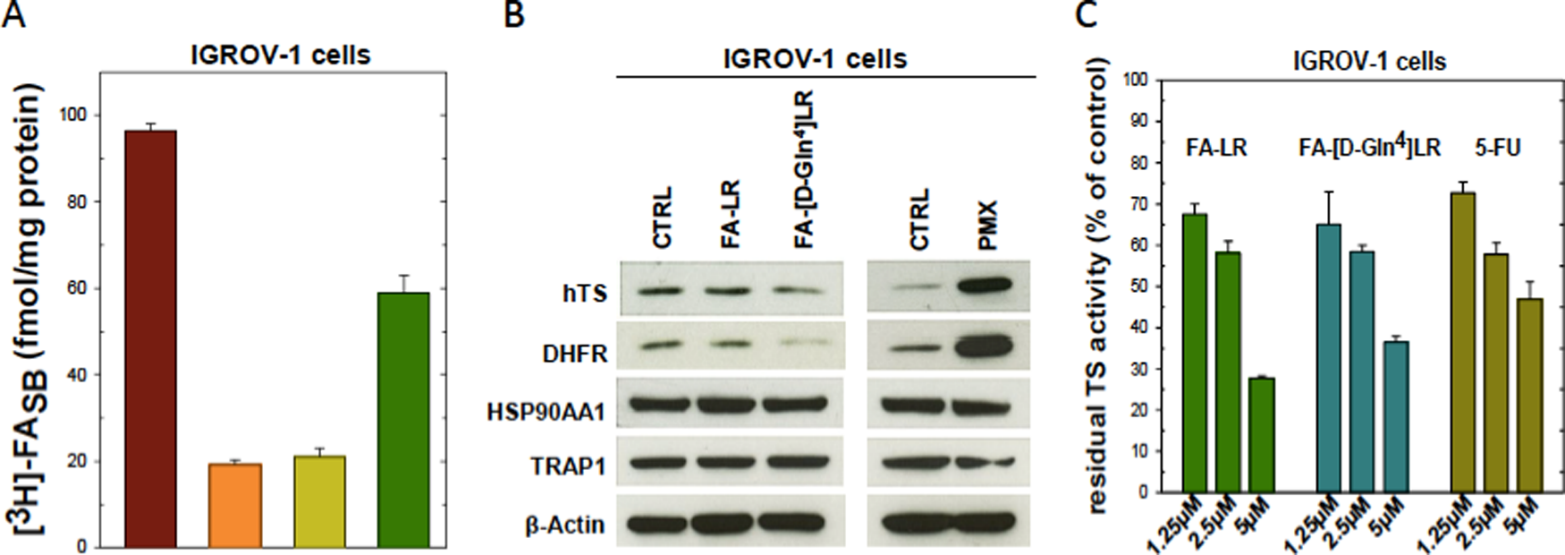
(A) Femtomoles of [^3^H]FA bound to the IGROV1 cell surface per mg of protein ([^3^H]FASB) with the tritiated ligand administered alone (brown) and in the presence of cold FA (orange), FA–LR (light green), or the FA–[DGln^4^]LR conjugate (dark green) (see the Experimental Procedures section for details). (B) Immunoblot quantitative analysis of hTS, DHFR, HSP90AA1, and TRAP1 in IGROV-1 cells with FA–LR, FA–[DGln^4^]LR, and pemetrexed (PMX) after 48 h from treatment. The representative blots of three independent experiments are shown. Human β-actin was used as internal control for protein loading. The quantitative results, obtained by densitometric scanning of the protein blots, are plotted in Figure SI-11. (C) Inhibition of intracellular TS activity by the FA–peptide conjugates and 5-FU in IGROV-1 cells. All of the data plotted represent the mean of three separate experiments performed in duplicate. Error bars, S.E.M.

The capability of both conjugates to bind FRα with comparable affinities (Figure 6) is confirmed by the results of the binding assays performed toward FRα with FA–LR at a fixed concentration, 5 μM (Figure 9A). We determined displacements of [^3^H]FA from IGROV-1 cell surfaces of 80% and 40% by FA–LR and FA–[DGln^4^]LR, respectively. From standard coupled equilibrium analysis, we can see that these figures correspond to a ratio of the FRα/FA–peptide binding equilibrium constants near 5, which correspond to a difference between the Δ*G*°s only around 4 kJ/mol, consistent with the close resemblance of the FRα-binding modes of the two conjugates suggested by the docking simulations.

We finally turn to the effects of the two FA–peptide conjugates on the expression of hTS and the other proteins of a panel previously identified to specifically characterize the intracellular activity of the LR-type peptides^50^ (Figures 9B, SI-10, and SI-11). IGROV-1 cell growth inhibition (Figure 8) correlates with inhibition of intracellular TS expression and activity (Figure 9B,C).

To mark the mechanistic differences, the effects of the two FA–peptide conjugates that are expected to bind at the protein dimeric interface were compared, always at a 5 μM concentration, with those of pemetrexed (PMX) and 5-FU, classical anticancer compounds directed to the TS active site. The hTS protein levels were reduced by about 20% by FA–[DGln^4^]LR, but they were 2.5-fold upregulated by PMX (Figure 9B) and slightly increased by 5-FU (Figures SI-10 and SI-11). Moreover, we observed a clear decrease of the levels of the DHFR protein with respect to control after FA–LR and FA–[DGln^4^]LR treatment, as also previously observed in cells transfected with LR and [DGln^4^]LR.^8,9^ The other two proteins were only minimally affected. In particular, the level of the heat shock protein HSP 90-α (HSP90AA1) was reduced by only 10% by FA–[DGln^4^]LR, whereas the levels of the heat shock protein 75 kDa and the mitochondrial (TRAP1) levels were downregulated by approximately 20% by the two conjugates. The results indicate that the two FA–peptide conjugates modulate the hTS, DHFR, HSP90AA1, and TRAP1 protein panel, previously identified as an activity marker for the present class of compounds, in a similar way to the LR and [DGln^4^]LR peptides, thus supporting the conclusion that the conjugates maintain the same intracellular mechanism of action, TS-protein targeting included.

The TS activity, measured by the tritium release assay, decreased by approximately 30 and 40% at, respectively, 1.25 and 2.5 μM, for both FA–peptide conjugates (Figure 9C); these inhibition extents are comparable with those caused by 5-FU at the same doses. Possibly because of the contribution from an additional internalization mechanism (e.g., via the RFC), at 5 μM the effect of the conjugates was quite strongly enhanced, leaving only about a 30–35% of residual TS activity versus the 50% left by 5 μM 5-FU (Figure 9C).

### Growth Inhibition Combination Studies of LR with Anticancer Drugs

FA–LR can enter cancer cells that overexpress FRα at a low concentration, in the 100–500 nM range, and causes a 35% inhibition of cancer cell growth. We have investigated the effect on growth inhibition of combinations of FA–LR with known anticancer drugs that, alone, cause hTS overexpression by evaluating the synergism quotients (SQ). Three anticancer drugs were selected for the combination experiments, namely, cisplatin (cDDP), raltitrexed (RTX), and 5-fluorouracil (5-FU). It has been reported that in ovarian and other cancer types resistance to Pt-drugs is associated with high levels of hTS and cross-resistance to the hTS inhibitors 5-FU and RTX. The latter was demonstrated in preclinical cancer cell studies,^51−54^ and it was possible to correlate the in vitro results with the clinical data.^55,56^

We have recently shown that the proper drug combination sequence of cDDP:LR, RTX:LR, and 5-FU:LR, where LR was wrapped into a specific peptide delivery system or encapsulated into PEGylated pH-sensitive liposomes, was able to counteract resistance to cDDP and anti-hTS drugs. In particular, we observed that the simultaneous treatment or 24 h pretreatment of cells with the peptide followed by either agent produced synergistic effects even in resistant cells.^57^

**Figure 10:**
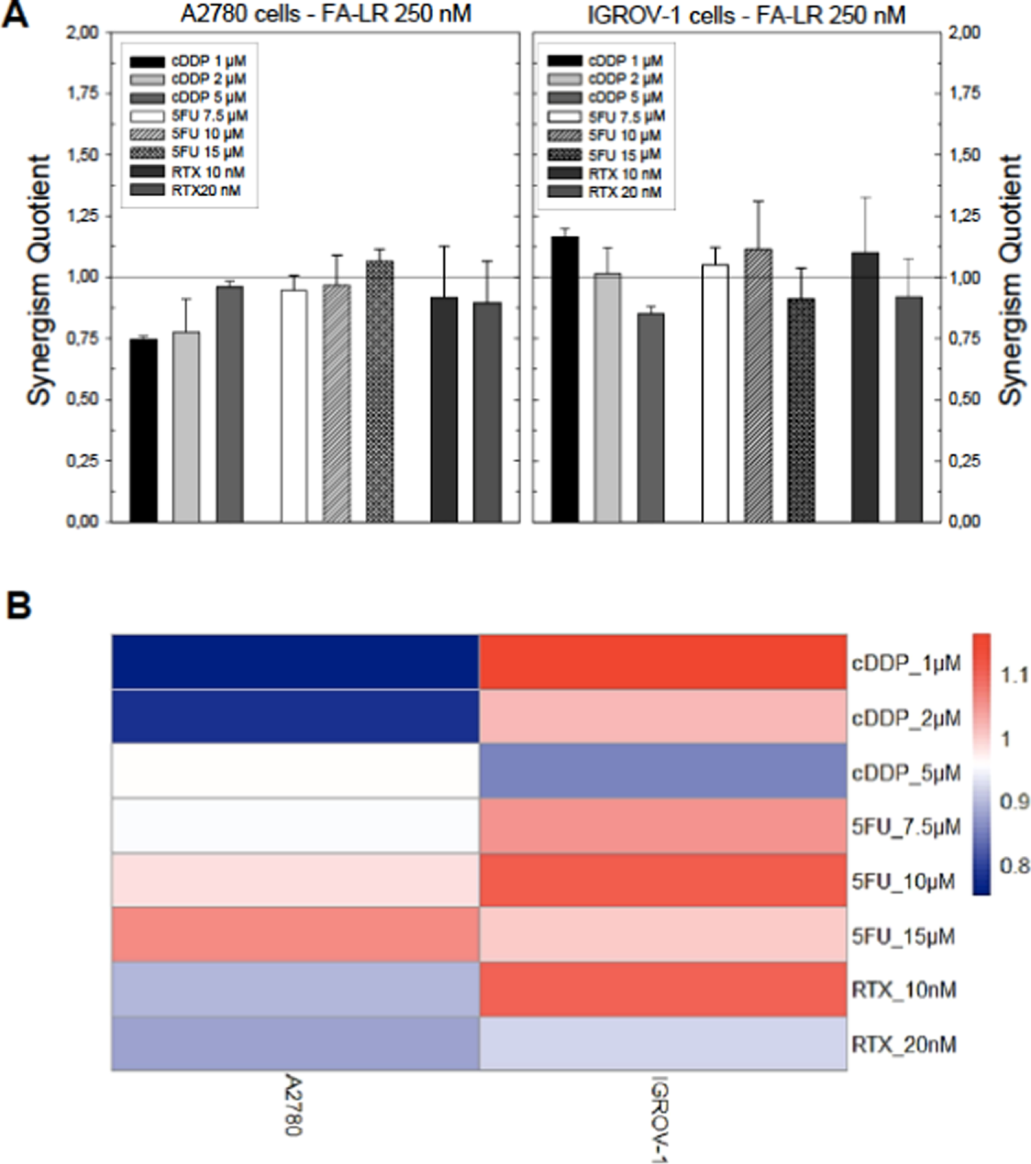
(A) Synergism quotients (SQ, ratios of the inhibition of a drug combination to the sum of the inhibitions of the two drugs alone) obtained in combination experiments of FA–LR with cDDP, 5-FU, and RTX on A2780 and IGROV-1 cells. SQ > 1.1, synergism; 1.1 > SQ > 0.9, additivity; SQ < 0.9, antagonism. The bars represent the mean of duplicate cell counts on three or more separate experiments. Error bars, SD. (B) Heat map representation of the SQ values of the tested combinations (rows) of FA–LR bioconjugate with cDDP, 5-FU, and RTX against the A2780 and IGROV-1 cell lines (columns). The reported dendrogram was obtained based on the dissimilarity matrix using Euclidean distances and the complete linkage method.

In the present study, FA–LR is combined with concentrations of RTX in the low nanomolar range (10–20 nM), with 5-FU in the 5–15 μM range and cDDP in the 1–5 μM range. FA–LR, internalized at 250 nM by exploiting FRα-induced endocytosis, demonstrated some synergistic combination effects. The best results were obtained with IGROV-1 cells that overexpress FRα. As shown in Figure 10A,B and Table SI-8, with A2780 cells the combination of FA–LR and cDDP at the three concentrations tested produced effects from antagonism to additivity, with SQ values from 0.75 to 0.96, as the concentration of cDDP was increased. The opposite was observed with IGROV-1 cells, from a slight synergism (SQ = 1.16) to additivity (SQ = 1.02) and then antagonism (SQ = 0.85) as the concentration of cDDP increased. In any case, the results confirmed that this combination resulted in enhanced cell growth inhibition, from 37.9 to 87.9% with the A2780 cells (Table SI-8) and from 40.3 to 63.20% with the IGROV-1 cells. A similar trend, but with smaller variations, was observed for the three combinations of FA–LR with 5-FU and RTX, also evidenced by the deep red colors of the heat map in Figure 10B in which the SQ values of all of the tested combinations are shown with the appropriate statistical analysis (see the Experimental Procedures section). The relative percentages for each combination are reported in Table SI-8. Overall, the combination studies provided at least four and three additive cell killings with A2780 and IGROV-1 cells, respectively. Noteworthy, the best results, with two synergistic SQ values, were obtained with the FRα-overexpressing IGROV-1 cells, thus indicating the potential of these combinations for targeting cancer cells with higher FRα levels.

## Conclusions

The FRα protein, which is highly expressed in ovarian cancer cells, provides the opportunity for a targeted drug delivery and therefore for a major therapeutic advancement. In the present work, we have combined hTS unconventional inhibition with targeted delivery by designing an FA–peptide investigational lead candidate. We have synthesized two such conjugates, FA–LR and FA–[DGln^4^]LR, have explored their affinity for FRα in cancer cells with different levels of this protein, have investigated their inhibition mechanism versus recombinant hTS, have evaluated their concentrations in cancer cells and their effects on cell growth as single agents and in combination with other anticancer drugs. Finally, we have traced their intracellular TS engagement by measuring their ability to inhibit the protein activity in cells and by analyzing their modulation of the levels of the proteins of a set that represents a specific cellular marker of these hTS dimer interface binding peptides.

Both conjugates inhibited recombinant hTS with a mixed/almost noncompetitive mechanism that can be interpreted in terms of a dual binding mode, in keeping with their difunctional nature. However, binding of the FA peptides at the monomer–monomer interface of hTS was found to be characterized by affinities at least 1 order of magnitude larger than those for binding at the catalytic site. Our competition experiments have shown that FA–LR binds FRα of cell membranes with an affinity comparable with that of FA. The competing effect of FA–LR on FA uptake is more pronounced with cells, like IGROV-1 and OAW28, that express high levels of FRα, thus suggesting a selectivity of the FA–peptide conjugates for cell lines with high levels of this receptor. The two FA–peptide conjugates showed a cell growth inhibitory activity comparable with that of 5-FU, an active-site-directed TS inhibitor. The FA conjugates/TS engagement was relevant to the observed cytotoxicity as suggested by both the inhibition of intracellular hTS activity and by the observed modulation of a protein set that is a specific intracellular marker of the activity of these peptides. In particular, following treatment with the two FA–peptide conjugates, the cellular levels of hTS were found almost unmodified or slightly downregulated and DHFR was downregulated. This suggests a cellular mechanism of action similar to that of the peptidic fragments, i.e., a mechanism based on interaction with the protein monomer–monomer interface.

To quantify the FA–LR peptide internalized into cancer cells, we have developed two independent approaches. We have employed the fluorescence of the pteroate anion resulting from UV-light-assisted hydrolysis of FA in basic environments. Because it relies on pteroate fluorescence, this label-free fluorometric method might be extended to a wide range of FA–drug conjugates and, more importantly, it could be directly applied to studies of FA–drug conjugate pharmacokinetics in clinical samples, if the conjugates are not metabolized. The intracellular concentration estimates obtained in this way were confirmed by a high-sensitivity LC-MS/MS analysis, that focused on compartmentalization of the compound, and an LC-MS Orbitrap approach that allowed us to evaluate the biostability in the cytosolic environment over time. The latter was characterized by a half-life of 200 min at 37 °C.

FA conjugates can selectively enter cells that overexpress FRα, ruling out healthy cells, in the low hundreds of nanomolar range (100–250 nM). Therefore, at these concentrations, they have the potential to be developed into a new tool for cancer chemotherapy with low toxicity. Because of their peculiar allosteric mechanism of action, they are expected to act as selective inhibitors that can be combined with chemotherapeutic agents and, by avoiding the increase of hTS levels, can hinder drug resistance development.

FA–LR was engineered as a conjugate with suitable chemical stability. The intracellular degradation of FA–LR releases the LR peptide as the most relevant metabolite (data not shown), i.e., an hTS inhibitor. On the other hand, should a similar degradation occur outside cells, in vivo, the inability of this peptide alone to be internalized by cells without a suitable delivery system,^8,12,13^ makes this event unlikely and the ensuing toxicity effects probably negligible. Furthermore, as we propose to use a combination of the FA-conjugate with an anticancer drug and expect that the conjugate concentration in the combination is as low as a few hundred nanomolars, the problem of the toxicity of the individual agent is further minimized.

This work has finally demonstrated that the FA peptides, once combined with cDDP, RTX, and 5-FU, can help overcoming resistance toward these drugs, developed in OC cells, particularly in highly FRα-overexpressing ones. This may help increasing the therapeutic potency and reduce toxicity of such drugs. With respect to the previously proposed treatment with peptides delivered with a nonliposomal peptide delivery system, these conjugates represent a step forward in the direction of selectivity.

Our future work will focus both on the improvement of the intracellular trafficking of the new conjugates in cancer cells and on their evaluation in experimental conditions in which their potential selectivity for FRα (upregulated on tumor cells) over FRβ (upregulated on activated monocytes and macrophages) might be exploited in cancer tissues in the presence of inflammation.

The association of standard drugs with the peptidic FA-bioconjugate hTS inhibitors may have the potential for future clinical applications to overcome the drug resistance to cDDP and anti-hTS drugs in ovarian tumor patients.

## Experimental Procedures

### Synthesis of the FA–LR and FA–[DGln^4^]LR Conjugates

The fragments [γGlu^0^]LR-resin and [γGlu^0^, DGln^4^]LR-resin were synthesized in accordance with a previously published methodology.^7^ The product, [γGlu^0^]LR or [γGlu^0^, DGln^4^]LR, linked to the resin (100 mg, loading: 0,445 mmol/g) was suspended in DMF (3 mL) and reacted with *N*^10^-(trifluoroacetyl)pteroic acid (2.0 equiv, 36 mg), 1-[bis(dimethylamino)methylene]-1*H*-1,2,3-triazolo[4,5-b]pyridinium 3-oxide hexafluorophosphate (HATU) (2.0 equiv, 34 mg) and *N*,*N*-diisopropylethylamine (DIPEA) (3.0 equiv, 15 μL). The reaction was slowly stirred at room temperature for 18 h after which the solvent was removed, and the resin was washed with DMF (3 × 5 mL) and CH_2_Cl_2_ (3 × 5 mL). The on-resin bioconjugates were suspended in DMF (3 mL) and a solution of 20% NH_2_NH_2_ in DMF (1 mL) was added to remove the trifluoroacetyl moiety. The reaction was slowly stirred at room temperature for 30 min, then the solvent was removed, and the resin was washed with DMF (3 × 5 mL) and CH_2_Cl_2_ (3 × 5 mL). Finally, the peptide bioconjugate was cleaved from the resin with reagent B (trifluoroacetic acid/H_2_O/phenol/triisopropylsilane 88:5:5:2; v/v; 10 mL/0.2 g of resin) for 1.5 h at room temperature. After filtration of the exhausted resin, the solvent was concentrated in vacuo and the residue was triturated with Et_2_O. After complete evaporation of the solvent, FA–LR and FA–[DGln^4^]LR were purified by preparative reversed-phase high-performance liquid chromatography (HPLC) and fully characterized by analytical HPLC and mass spectrometry (Supporting Information). Purity is >95%.

### Molecular Modeling

FA–LR and FA–[DGln^4^]LR were docked in hTS and FRα with the GOLD software, version 5.2.2 (www.ccdc.cam.ac.uk). The structure of FRα complexed with FA (PDB code 4LRH) was used as a template for docking the conjugates in FRα. The structures of hTS complexed with dUMP and raltitrexed and with the LR peptide (PDB code 1HVY and 3N5E, respectively) were used for docking the conjugates in the active and inactive forms of the protein. For each compound, 25–50 diverse poses were generated and analyzed. A radius of 15 Å was set when targeting the hTS and FRα binding sites, while 30 Å were allowed when placing a ligand at the hTS protein subunit interface. A maximum number of 100 000 operations were performed for each docking search, on a population of 100 individuals with a selection pressure of 1.1. Operator weights for crossover, mutation, and migration were set to 95, 95, and 10, respectively. The number of islands and the niche were set to 5 and 2. Hydrogen bond constraints were imposed when targeting the hTS and FRα binding sites. Flexibility of crucial residues lining the binding sites and the monomer–monomer interface was allowed. The default GoldScore fitness was used as native scoring function.^58^ Docking at the hTS interface was run with and without distance constraints, to place cysteine 3 at a suitable disulfide bond distance from Cys125, monomer 1. Similar peptide positions were obtained in the two cases.

### Enzyme Inhibition Assays

The peptides and their folic conjugates were tested against the recombinant hTS protein spectrophotometrically using a SpectraMax 190 microplate reader (Molecular Devices). The enzymatic reaction was monitored spectrophotometrically by measuring the absorbance (A) at 340 nm for 180 s. Each peptidic inhibitor was assayed at the concentrations reported in Figure 3 following a 1 h incubation with the target enzyme at 37 °C with gentle orbital shaking (60 rpm). The initial slope of each *A*_340_/time plot was employed to compute the initial reaction rate (*v*). The *v* values were then analyzed as functions of the mTHF cofactor concentration at each inhibitor concentration as discussed in the Mechanism of Inhibition of Recombinant hTS section. Each inhibition assay was performed in triplicate for calculating an error value with a 95% confidence interval (*p* ≤ 0.05). Additional experimental details are provided in the Supporting Information.

### Cell Lines, Cell Growth Inhibition, and Intracellular TS Activity

The human OC cell lines OAW28, COV504, IGROV1, TOV112D, 2008, C13*, A2780, and A2780/CP include serous, endometrioid, clear cell, and mixed-type cell lines, as well as for the sensitivity to cisplatin.^33,34^ They were grown as monolayers in Roswell Park Memorial Institute (RPMI) 1640 medium containing 10% heat-inactivated fetal bovine serum (FBS) and 50 μg/mL gentamycin sulfate. All cell media and serum were purchased from Lonza (Verviers, Belgium). Cultures were equilibrated with humidified 5% CO_2_ in air at 37 °C. Before each experiment, the cells were pretreated with folate-free (FF) RPMI 1640 medium (pH 7.2) for 24 h to allow the externalization of FR on cell surface.

Cell growth was determined using a modified crystal violet dye assay.^59^ And 24 h after seeding in complete medium, this was replaced with FF medium and the cells were treated with increasing concentrations of the bioconjugates and 5-FU three times every 12 h. Then, the cells were allowed to grow up to 72 h. TS activity was measured in extracts from cells treated in the same conditions as used in the cytotoxicity experiments. TS catalytic assay^35^ is based on the measurements of the amounts of ^3^H release from 5-[^3^H]dUMP during its TS catalyzed conversion to dTMP. Briefly, the reaction was started by adding 5-[^3^H]dUMP (1 μM final concentration, specific activity 5 mCi/mol) to enzyme suspensions in assay buffer and 650 μM 5,10-methylenetetrahydrofolate in a final volume of 50 μL. After incubation for 60 min at 37 °C and blocking by adding 50 μL of ice-cold 35% trichloroacetic acid, residual 5-[^3^H]dUMP was removed by the addition of 250 μL of 10% neutral activated charcoal. The charcoal was removed by centrifugation at 14 000*g* for 15 min at 4 °C, and a 150 μL sample of the supernatant was assayed for tritium radioactivity in a liquid scintillator analyzer Tri-Carb 2100 (Packard).

### Real-Time PCR of FRα mRNA

Cells were harvested by scraping and total RNA was isolated using the InnuSOLV RNA reagent (Analytik Jena, Germany). Reverse transcription was performed essentially as previously reported.^60^ We performed dissociation curve analysis and agarose gel electrophoresis to confirm the amplification. The amount of RNA expressed was normalized with GAPDH and detected by 2^–ΔCt^ method. FOLR1 (target) primers [NCBI, CoreNucleotide: NM_016725.2]: forward: 5′ GTGAGCAATGGTGGGAAGAT 3′, reverse: 5′ GTGGGTGTGGGGAAGTAGAA 3′; GAPDH (reference) primers [NCBI, CoreNucleotide: NM_002046.3]: forward: 5′ CAAGGTCATCCATGACAA CTTTG 3′,reverse: 5′ GGGCCATCCACAGTCTTCTG.

### Western Blot Analysis

For the assessment of enzyme levels, 40 μg of cellular proteins were resolved by sodium dodecyl sulfate-polyacrylamide gel electrophoresis (SDS-PAGE). Western blot analysis of TS and DHFR was conducted as previously described,^61^ using a 1:250 dilution of the anti-human TS mouse TS106 monoclonal primary antibody (Abnova, Italy), and 1:250 dilutions of the anti-human DHFR mouse A-4 monoclonal antibody (Santa Cruz Biotechnology, Inc.). Cells were plated in complete medium containing 10% heat-inactivated FBS and after 24 h, in FF medium (pH 7.2), except for the control sample (CTRL). After an additional 24 h, the cells were treated with the FA–peptide conjugates or PMX. For the assessment of FRα levels, nonreducing and nondenaturating conditions (no SDS) were used. Gels were blotted onto poly(vinylidene difluoride) (PVDF) membranes (Hybond-P, Amersham). Antibody staining was performed with a chemiluminescence detection system (ECL Plus, Amersham), using a 1:500 dilution of the anti-human mouse Mov18 monoclonal primary antibody (Enzo Life Sciences) and 1:2000 dilution of anti-human β-actin mouse AC-15 antibody (Santa Cruz Biotechnology, Inc.) in TBS-T with 5% dry milk for normalization, in conjunction with a 1:1500 dilution of a horseradish peroxidase-conjugated sheep anti-mouse secondary antibody (Amersham).

### Flow Cytometric Analysis of FRα Cell Surface Expression

The Mov18 primary antibody (10 μg/mL, Enzo Life Sciences) was added to tumor cells (3 × 10^5^) in 100 μL of phosphate-buffered saline (PBS) + 1% bovine serum albumin, and the mixture was incubated for 1 h at room temperature. The antibody binding was detected by an fluorescein isothiocyanate (FITC)-conjugated secondary antibody (1:200, Dako) for 30 min at 4 °C in the dark. For each sample, at least 10 000 cells were acquired with an Epics-XL flow cytometer (Beckman Coulter) and data were subsequently analyzed using the WinMDi software.

### Radioligand Assays

To assess FRα at the cell surface, cells were incubated for 10 min with 5 nM [^3^H]FA (specific activity 0.5 Ci/mmol) in ice-cold PBS (pH 7.4) in the presence or absence of 5 μM unlabeled FA or FA–LR (1000-fold in excess of [^3^H]FA), and incubated for 10 min at 4 °C.^35^ Uptake studies were conducted with 30 nM [^3^H]FA at 37 °C in the presence and absence of 10 μM unlabeled FA or FA–LR conjugate.^62,63^

### Fluorometric FA–LR Assay

Uptake of FA–LR was spectrofluorometrically measured following incubation of ca. 6 × 10^6^ IGROV1 cells with PBS (pH 7.4) in the presence and absence of 5 μM FA–LR at 37 °C for 20 min, three homogeneous samples each. Cell extracts had a final volume of 700 μL. NaOH (2 μL, 1 M) was added to each solution to a pH of around 11, to convert all pterins to pteroate fluorophores. Emission (λ_exc_ = 360 nm) and excitation (λ_em_ = 470 nm) spectra were measured on a Horiba FluoroMax2 spectrofluorometer in 4 × 10 mm^2^ quartz cuvettes at room temperature (25–28 °C). Because irradiation caused an increase in the pteroate fluorescence intensity, likely associated with photocleavage of the pteroyl/*p*-aminobenzoate bond,^44^ emission/excitation spectral measurements were repeated until the spectra did not change in two subsequent measurement cycles. Pteroate emission was read at 445 nm and excited at 360 nm. To obtain the calibration factors needed to convert these signals into pteroate concentrations, the same measurements were carried out on the three control samples following additions of known, increasing amounts of the 5 μM FA–LR supernatant.

### LC-MS Analysis to Study FA–LR Intracellular Compartmentalization

IGROV-1 human ovarian cancer cells were cultured in RPMI medium supplemented with 10% fetal bovine serum (FBS) plus 20 mM l-Gln at 37 °C with 5% CO_2_. RPMI medium was aspirate and replaced by FF-RPMI medium (folate-free RPMI medium) to induce overexpression on the folate receptor on cell surface. After 24 h, FF-RPMI medium was aspirated and replaced by a solution of FA–LR in 5 μM PBS. After 30 min from the FA–LR delivery, samples were collected to detect the concentration of FA–LR in the extracellular medium. The compound was detected to be stable in PBS in the first 30 min. Cells were exposed to the conjugate solution for 30 min at both 37 and 4 °C. About 2 million cells were washed three times with acid PBS (pH 5.0), scraped, and counted. Hypotonic lysis buffer (30 μL) was added to each cell pellet and incubated at room temperature for 1 h. Three freeze–thaw cycles were performed to lysis cell membranes. Afterward, to collect separately vesicles and cytosol, the samples were centrifuged at 14 000 rpm for 30 min at 4 °C and their supernatants were pipetted into 1.5 mL Eppendorf tubes. Acetonitrile (ACN) was added to the supernatant aspirated from the pellets to obtain 1:1 v/v with the hypotonic buffer solution, while a mixture of ACN/H_2_O (50:50, v/v) was added to the pellets. Both supernatants and pellets were centrifuged at 14 000 rpm for 30 min at 4 °C.^39^ Finally, IS at a final concentration of 1 μg/mL was added both to the pellets and supernatants, and they were centrifuged at 14 000 rpm for 25 min. The amount of peptide in both pellets and supernatants was determined by LC-MS/MS analysis using an Agilent HP 1200 HPLC coupled to an Agilent 6410 triple quadrupole mass spectrometer, working in selected reaction monitoring (SRM) mode.^64^ The presence of FA–LR was studied through bi-charged (717.2 *m*/*z*) and tri-charged (478.5 *m*/*z*) parent ions, with both a qualifier and a quantifier daughter ion peak for each MS/MS transition. IS peak pattern was acquired in the same way. Before sample analysis, a qualitative–quantitative method validation was performed.

### Liquid Chromatography–Mass Spectrometer (Orbitrap Q-Exactive LC-HRMS) to Detect FA–LR Cytosolic Stability

C13* human ovarian cancer cell lines (highly cisplatin-resistant) were prepared as reported for IGROV1 (LC-MS compartmentalization experiment).^65^ Cells were incubated for five different time lapses in Petri plates: 20, 40, 60, 120, and 240 min. After each interval, the cells (about 2 million per Petri plate) were washed three times with acid phosphate-buffered saline (PBS, pH 5.0), scraped, and counted. Hypotonic lysis buffer (30 μL, pH 8, 20 mM *N*-(2-hydroxyethyl)piperazine-*N*′-ethanesulfonic acid (HEPES), 10 mM KCl, 1.5 mM MgCl_2_, 1 mM ethylenediaminetetraacetic acid (EDTA), 250 mM sucrose, 0.1 mM cOmplete Protease Inhibitor) was added to each cell pellet and incubated at room temperature for 1 h to perform osmotic lysis of cell membrane. The cells were centrifuged at 14 000 rpm for 30 min, and their supernatants—the cytosolic fractions—were pipetted into 1.5 mL Eppendorfs. Each cell supernatant was added with 100 μL of a 5 μM acetonitrile solution of internal standard (ISTD), an FA–LR-like octapeptide. Each cytosol aliquot was diluted to a final volume of 500 μL with a 1:1 mixture of H_2_O with 0.1% HCOOH and ACN with 1 μM 1,4-dithiothreitol (DTT) as an antioxidant agent.

#### Sample Analysis

Sample analysis was carried out on an UltiMate 3000 UHPLC (Thermo Fisher Scientific) coupled to an Orbitrap Q-Exactive mass spectrometer. We used an Agilent Poroshell C18 column (120 Å, 100 × 2.1 mm^2^, 2.7 μm ps) to separate the analytes, thermostated at 30 °C, with an injection volume of 20 μL per sample. Chromatographic profile started with 98% aqueous phase with 0.1% HCOOH (A) and 2% ACN (B). At 13 min, solvent B was raised to 28%, and at 16 min to 95% until min 20. Using tSIM mode in positive electrospray ionization (ESI) source, FA–LR as [M + 3H]^3+^ with a *m*/*z* of 478.5515 (rt. 12.44), IS as [M + 2H]^2+^ with a *m*/*z* of 444.2168 (rt. 12.16), together with the exact masses of “free” LR octapeptide have been incorporated in the inclusion list. Spectrometer parameters set up to maximize FA–LR signal were: sheath gas, 40 au; auxiliary gas, 30 au; source temperature, 290 °C; sweep gas, 3 au; spray voltage, 3.5 kV; and capillary temperature, 320 °C. A regression curve was built preparing five different calibration solutions of FA–LR: 1, 5, 10, 50, and 100 nM concentrations, with 1 μM IS for each calibrator. Sample values (FA–LR areas divided by IS areas) were interpolated into regression curve (Figure SI-7) to obtain total sample concentration of the analyte, which was converted into single-cell cytosolic concentration. C13* cells were assumed to have a spherical volume of 2 pL, whose 60% represented by cytoplasm. Thus, interpolated values were multiplied by the final dilution value (500 μL) and divided by the number of cells for each sample multiplied by a single-cell cytosol volume (1.2 pL). This formula allowed us to quantify the total cytosolic concentration of FA–LR for a single cell.

### Synergism Analysis

The nature of the combination between FA–LR and drugs (namely, cDDP, 5-FU, or RTX) was quantified by synergism quotient (SQ).^66,67^ SQ was defined as the net growth inhibitory effect of the analogue combination divided by the sum of the net individual analogue effects on growth inhibition. A quotient of >1.1 indicates a synergistic effect, that between 0.9 and 1.1 indicates an additive effect, while a quotient of <0.9 indicates an antagonistic effect. A2780 and IGROV-1 cell lines were exposed to the combinations, and cell growth was determined using a modified crystal violet dye assay,^59^ as reported above. And 24 h after seeding in complete medium, this was replaced with FF medium and the cells were treated with 250 nM bioconjugate and drugs three times every 12 h until to complete 72 h from the treatment beginning. The heat map and clustering have been realized with the open-source software R and Bioconductor repository, using ggplot2 and Heatplus packages (https://cran.r-project.org/; https://www.bioconductor.org/).^68,69^ For the clustering (Euclidean distance, complete linkage clustering), to highlight the distance between antagonism, addition, and synergy values, the synergism quotient values were elaborated as follows: for synergism quotient values <0.9, a value of 10 was subtracted; for synergism quotient values ≥1.1, a value of 10 was added.

## Supporting Information Available

Description of detailed experimental data on HPLC purification and analysis of FA conjugates; enzymatic assay; molecular modeling of FA–LR and FA–[DGln^4^]LR at TS monomer–monomer interface; radioligand assay; LC-MS/MS analysis (compartmentalization); LC-MS/MS analysis (stability); fluorescence spectra of a lysate of IGROV1; study of FA–LR concentration changes with time using an Orbitrap Q-Exactive mass spectrometer; and cell survival of human ovarian cancer cell lines (showing low FRa expression) treated with peptide LR and its conjugate alone or transfected into cells by peptide delivery system (PDF)
Raltitrexedin1HVY.pdb (PDB)
LRin3N5E.pdb (PDB)
FALRin4lrh.pdb (PDB)
FALRin3n5e.pdb (PDB)
FALRin1HVY.pdb (PDB)
FAin4LRH.pdb (PDB)
FADGln4LRin4lrh.pdb (PDB)
FADGln4LRin3n5e.pdb (PDB)
Molecular formula strings (XLSX) (CSV)

## Supplementary Material

jm0c02107_si_001.pdf

jm0c02107_si_002.pdb

jm0c02107_si_003.pdb

jm0c02107_si_004.pdb

jm0c02107_si_005.pdb

jm0c02107_si_006.pdb

jm0c02107_si_007.pdb

jm0c02107_si_008.pdb

jm0c02107_si_009.pdb

## Abbreviations

FA: folic acid
FRα: folate receptor α
hTS: human thymidylate synthase
OCs: ovarian carcinomas
RFC: reduced folate carrier
SPPS: solid-phase peptide synthesis
WB: Western blot
