# Supplementary material for: Folic Acid–Peptide Conjugates Combine Selective Cancer Cell Internalization with Thymidylate Synthase Dimer Interface Targeting
Source: J Med Chem. 2021 Mar 12;64(6):3204–21. doi: 10.1021/acs.jmedchem.0c02107 (PMC8041318; doi:10.1021/acs.jmedchem.0c02107)
Supplement: Supplementary file 1 — jm0c02107_si_001.pdf [file jm0c02107_si_001.pdf]

SUPPORTING INFORMATION

Folic acid-peptide conjugates combine selective cancer cell internalization with thymidylate synthase dimer interface targeting.

Gaetano Marverti[a], Chiara Marraccini[b], Andrea Martello[a], Domenico D'Arca[a], Salvatore Pacifico[c], Remo Guerrini [c], Francesca Spyrakis[b,d], , Gaia Gozzi[a], Angela Lauriola[a], Matteo Santucci[b], Giuseppe Cannazza[b], Lorenzo Tagliazucchi[b] Addolorata Stefania Cazzato[b], Lorena Losi[b], Stefania Ferrari[b], Glauco Ponterini [b] and Maria P. Costi [b*].

[a] Dept. Biomedical, Metabolic and Neurosciences, Via Campi 287, University of Modena and Reggio Emilia; [b] Dept. Life Sciences, Via Campi 103, University of Modena and Reggio Emilia, 41125, Modena; [c] Dept. of Chemical and Pharmaceutical Sciences, Via Fossato di Mortara, University of Ferrara, Ferrara, Italy; [d] current address Dept. of Drug Science and Technology, Via Giuria 9, University of Turin, 10125, Italy.

Corresponding Author

* Maria P. Costi:

[b]Dept. Life Sciences, Via Campi 103, University of Modena and Reggio Emilia, 41125, Modena, Italy

Phone: 0039-059-205-5134; Fax: 0039-059-205-5131; email: mariapaola.costi@unimore.it

Table of content

HPLC Purification and analysis of FA-conjugates. pag. S3

Enzymatic Assay pag. S5

Molecular Modelling of FA-LR and FA-[DGln^{4}]LR at TS monomer-monomer interface pag. S8

Radioligand assay pag. S10

LC-MS/MS analysis (compartimentalization) pag. S10

LC-MS/MS analysis (stability) pag. S12

Fluorescence spectra of a lysate of IGROV1 pag. S19

Study of FA-LR concentration changes with time using Orbitrap Q-Exactive™ mass spectrometer. pag. S20

Cell survival of human ovarian cancer cell lines (showing low FR□ expression) treated with peptide LR and its conjugate alone or transfected into cells by peptide delivery system. pag. S22

HPLC Purification and analysis of FA-conjugates.

Purification of FA-conjugates were performed by using preparative reversed-phase HPLC: Water Delta Prep 3000 system with a Jupiter column C_{18} (250 x 30 mm, 300 A, 15 μm spherical particle size). The column was perfused at a flow rate of 20 mL/min with a mobile phase containing solvent A (5%, v/v, acetonitrile in 0.1% TFA), and a linear gradient from 0 to 60% of solvent B (60%, v/v, acetonitrile in 0.1% TFA) over 30 min for the elution of the conjugate. Analytical HPLC analyses were performed on a Beckman 116 liquid chromatograph equipped with a Beckman 166 diode array detector. Analytical purity of the compound was determined using a Luna C_{18} column (4.6 x 100 mm, 3 μm particle size) with the above solvent system (solvents A and B) programmed at a flow rate of 0.5 mL/min using a linear gradient from 0% to 80% B over 25 min. The desired products showed ≥ 95% purity when monitored at 220 nm (Figure SI 2a). The UPLC-MS analysis were performed by Waters Acquity UPLC equipped with a MICROMASS ZMD 2000 and using as column ACQUIY UPLC® BEH C18 column (2.1 x 50 mm, 1.7 μm particle size). The Figure SI 2b shows the UPLC chromatogram and MS spectrum of a FA-conjugate.

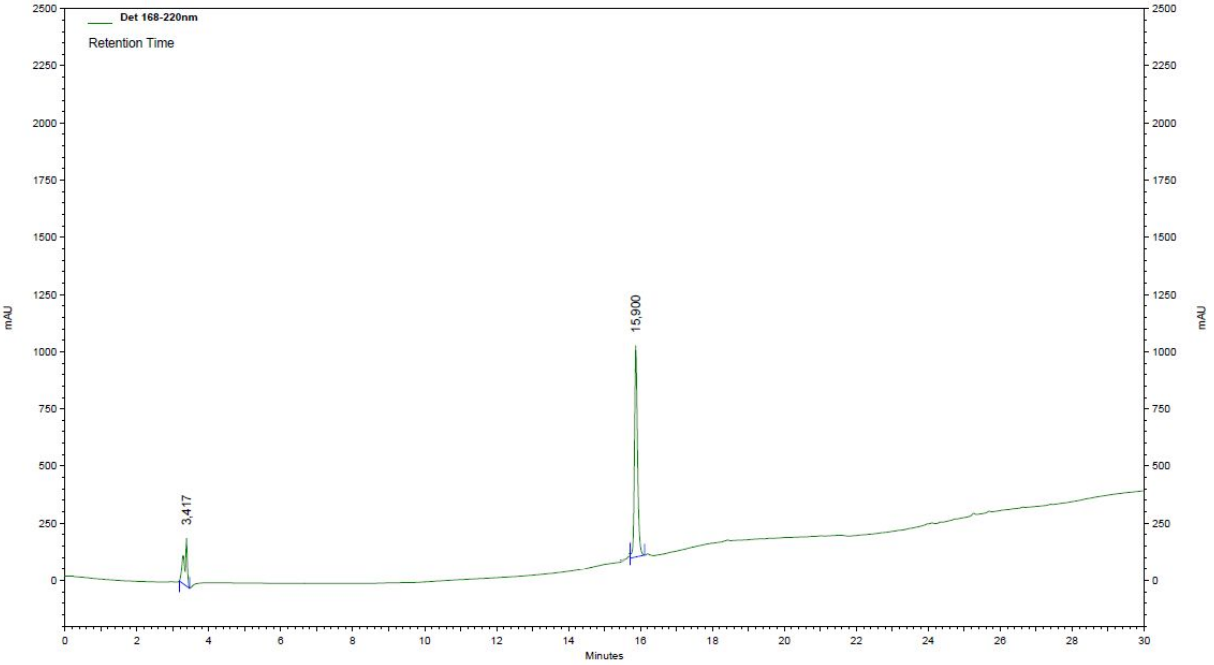

Supporting Figure SI-1a. Analytical HPLC profile of the conjugate compound FA-LR.

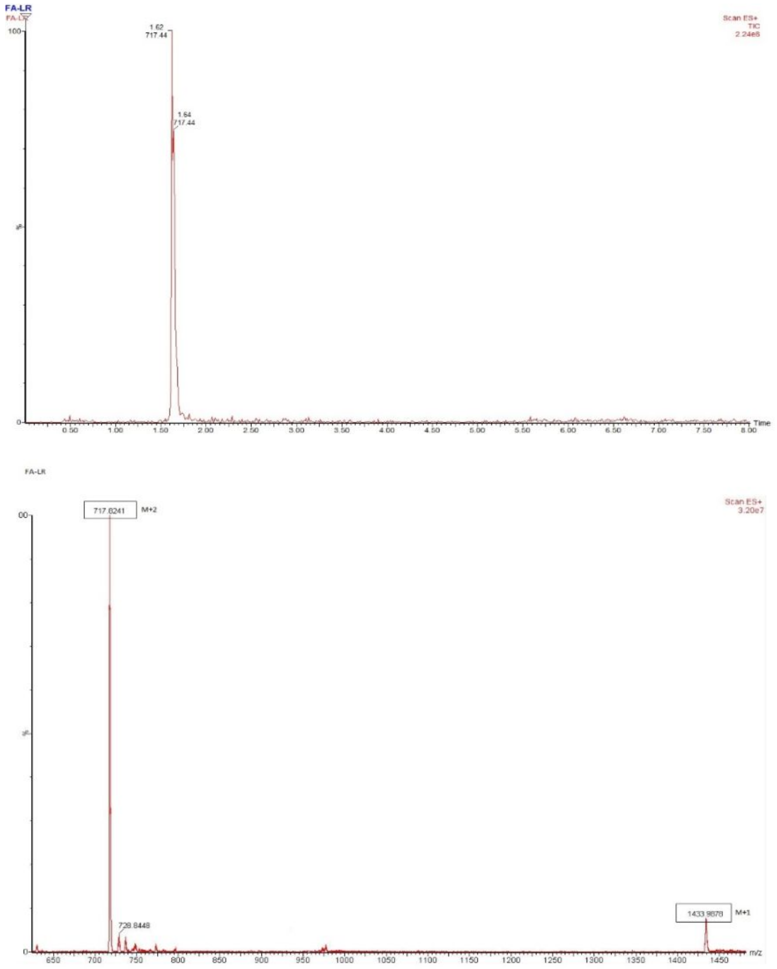

Supporting Figure SI-1b. UPLC-MS spectra of FA-[DGln4]LR

Enzymatic Assay.

hTS was purified as previously reported [1]. Enzyme solutions were thawed the day of the experiment and the enzyme concentration was determined by UV-Vis spectroscopy using ε_{280} = 89000 M^{-1} cm^{-1} and MW=74229 Da. Thawed protein solutions were kept constantly at 4°C. In this condition, enzyme was able to reproduce normal kinetic activity values ($K_{M\ dUMP}$ = 10-12 µM, $K_{M\ mTHF}$ = 4-6 µM, k_{cat} = 0,8 - 0,9 s^{-1}). The LR and [DGln4]LR peptides and the respective FA-conjugates were solubilized in bi-distilled water to prepare 10 mM stock solutions. The solutions were mixed or vortexed in order to help solubilisation and the concentration of each peptide solution was determined by UV spectrophotometry (ε_{280} = 1450 M^{-1} cm^{-1}). Each peptidic inhibitor was tested at the following concentrations: [LR] = 0, 25, 50 and 100 µM, [[DGln4]LR] = 0, 40, 80 and 150 µM, [FA-LR] = 0, 20 and 40 µM, [FA-[DGln4]LR] = 0, 50 and 75 µM. 3-5 mM stock solutions of mTHF were prepared in carbonate buffer ($NaHCO_3$ 30 mM) and the concentrations used during the Ki determination assays were in the 3.35 – 68.32 µM range. 10-12 mM stock solutions of dUMP were prepared in bi-distilled water and the concentration used in the assays was 140 µM, corresponding to saturation of the enzyme binding sites ([dUMP]/ $K_{M\ dUMP}$ = 12-14). An aliquot of recombinant hTS (2.5-3 µg) was added to 0.05 mL of assay kinetic buffer (50 mM TES, pH 7.4, 25 mM $MgCl_2$, 6.5 mM HCHO, 1 mM EDTA, 75 mM β-mercaptoethanol (β-ME)) and was incubated with a peptidic inhibitor at a given concentration for 1 hour at 37°C under gentle orbital shaking. Then, the remaining reactants of the kinetic assay were added to the reaction mixture as follows: mTHF in one of the concentrations above, dUMP 0.14 mM and bi-distilled water up to 0.1 mL. As a limiting-rate substrate, the dUMP was added last. From then on, the absorbance change was monitored at 340 nm in a UV-visible micro-plate spectrophotometer for 3 min. The slope at the origin was determined by using the following formula: $Rate = (A_s - A_{bl}) / (t_2 - t_1)$, (where A_s is the sample reading, A_{bl} is the blank solution reading subtracted from the corresponding sample reading at each time point, $t_2 - t_1$ are the limits of the time

interval for the best fit straight line) and yielded the initial reaction rate, v. The test was repeated 3 times (Carosati et al., 2012).

Superposition of FA-LR docked in TS active site with raltitrexed.

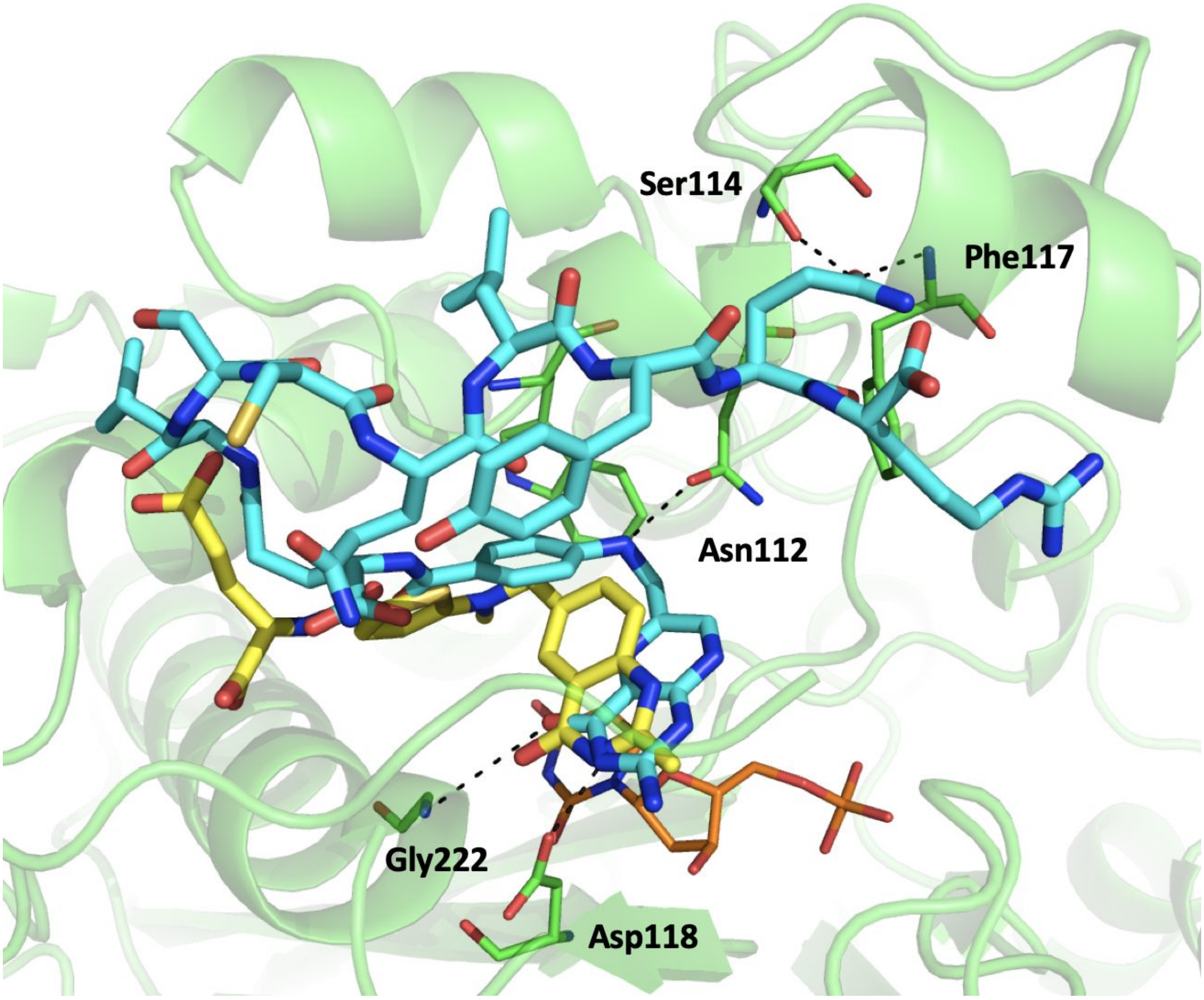

Supporting Figure SI-2.

Superposition of the docked conformation of FA-LR (cyan) with raltitrexed (yellow) co-crystallized with hTS (PDB code 1hvy). The protein is displayed in green cartoon while the ligands in capped sticks.

Molecular Modelling of FA-LR and FA-[DGln^4]LR at TS monomer-monomer interface.

The superimposition of the docking poses of FA-LR and FA-[DGln^4]LR with the structure of the LR peptide co-crystallized at the interface of hTS inactive form (PDB code 3n5e) is reported in Figure 4G and 4H, and shows a number of similarities among the predicted and experimental orientation of the peptide region.

In the case of FA-LR (Figure 4G and SI-3a), the orientation of Leu1 is well conserved, while the side-chain of Ser2 points in the opposite direction. A very closed position to the co-crystallized one is also maintained by Cys3, still located at a disulfide bridge distance with respect to the protein Cys180, and by Gln4, whose side-chain is well superimposed with the X-ray conformation. Major adjustments are, instead, underwent by the last three residues, i.e. Tyr6, Gln7 and Arg8. A slightly different orientation is assumed by the FA-[DGln^4]LR conjugate (Figure 4H and SI-3b). The side-chain of Leu1 lies at a short distance with respect to the crystallographic structure, as well as Cys3. On the contrary, the side-chain of both Ser2 and Leu5 point towards an opposite direction. Gln4 and Gln7 are shifted with respect to the X-ray orientation, while Tyr6 well resembles the original pose. Finally, as in the case of FA-LR, Arg8 assumes a completely different orientation with respect to the LR peptide. As previously underlined, in both cases the central part of the peptide region lies deeply into the crevice at the protein subunit interface while the folic moiety and the tail residues Tyr6, Gln7 and Arg8 occupy more solvent-exposed areas. The Goldscore Fitness scores for the two conjugates pose reported in Figure 4G and 4H were 79 and 89, respectively.

A detailed representation of the peptide orientation at the protein subunit interface and of the interactions made with the protein residues is reported in Supporting Figure SI-2.

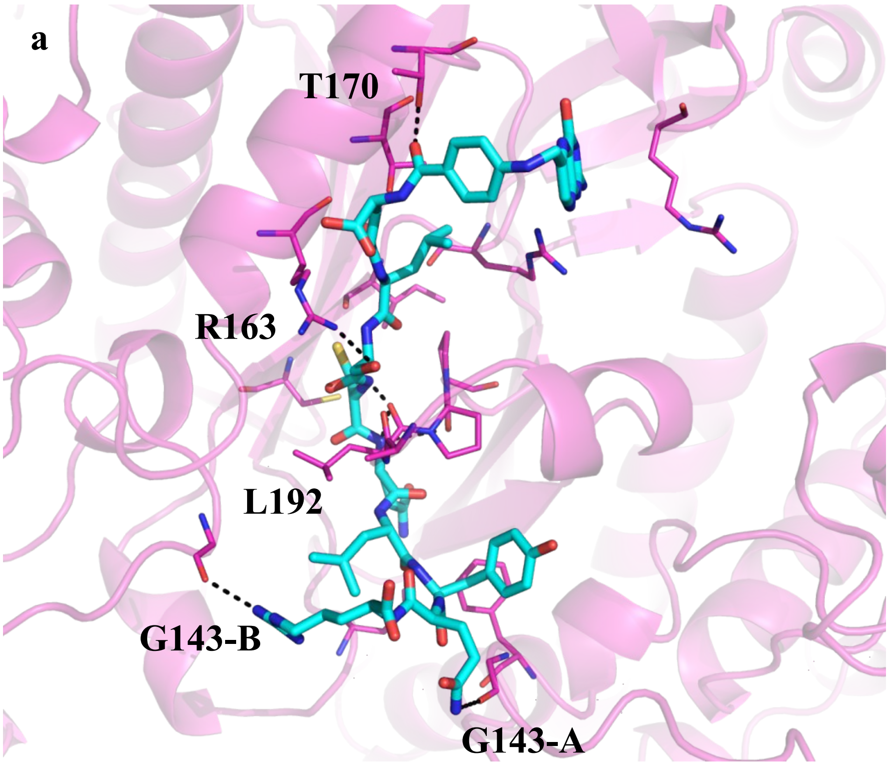
a
T170
R163
L192
G143-B
G143-A

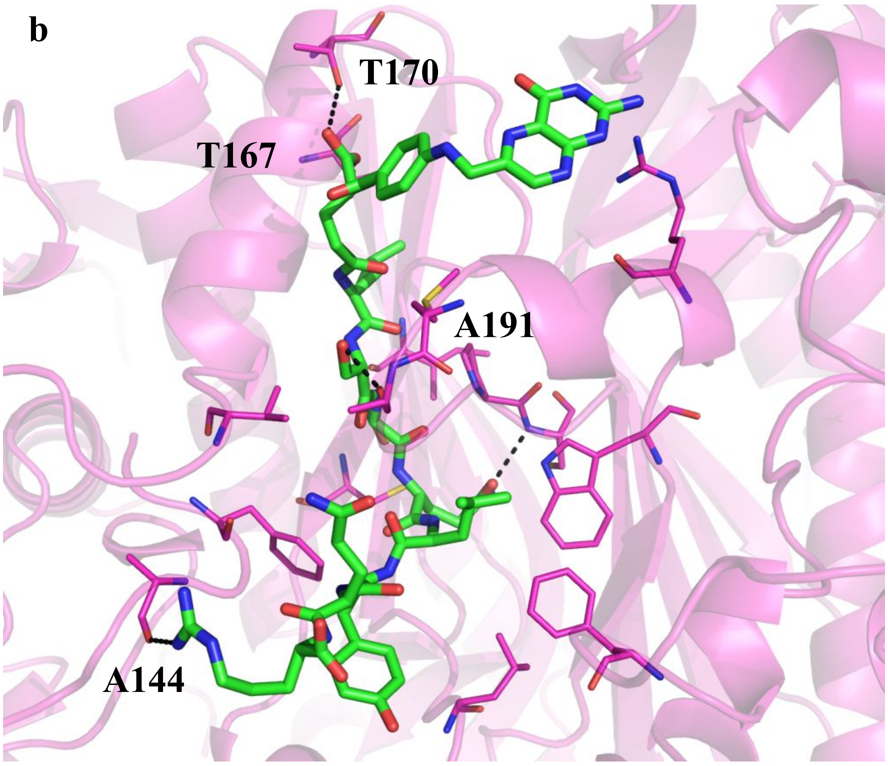
b
T170
T167
A191
A144

Supporting Figure SI-3. Docking pose of FA-LR (a) and FA-[DGln^4]LR (b) at the protein subunit interface of the hTS inactive form (IiI state; PDB code 3n5e)). The residues lining the pocket are represented as capped sticks. Those forming H-bonds with the peptides are labelled. H-bonds are shown as black dashed lines.

Radioligand assay

To accurately evaluate the functional FR protein levels, which is crucial for most FR-targeted therapies, we have also performed radioligand binding assays (Reddy et al., 1999). Once more, the IGROV-1 cells exhibited the highest amount of functional FR on their surface: more than 90 fmol of [^{3}H]folic acid bound on their surfaces per mg of protein, versus about 40 fmol/mg for OAW28 cells and values lower than 30 fmol/mg for the other cell lines (Figure SI-4).

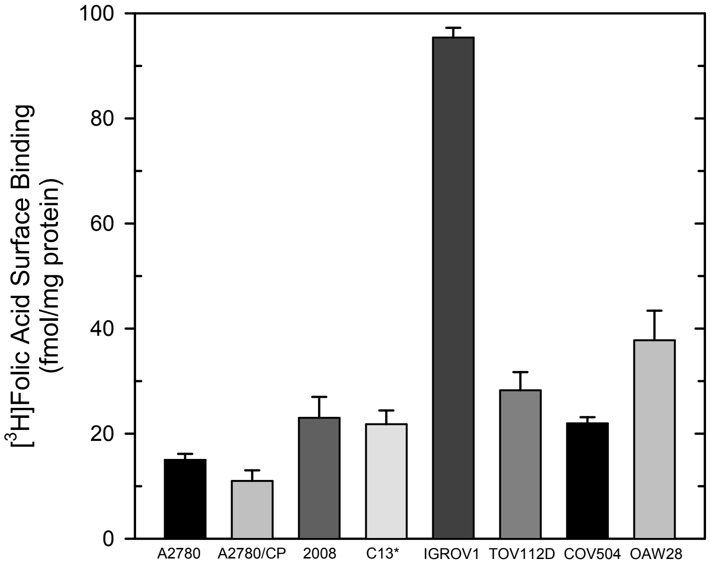

Supporting Figure SI-4. FRα expression as determined by [^{3}H]folic acid (5 nM) surface binding at pH 7.4 in the human ovarian cell lines. The level of occupied receptors was determined after

assessment of [^{3}H] folic acid binding at 4°C for 10 min. Each bar represents the mean ± S.E.M of three separate determinations.

By referring the concentration of FRα for amount of cellular biomass (protein content) with the cell number for each cell line, we obtained the number of receptors for single cell. It is thus calculated that the IGROV-1 cells have approximately 45x10^3 FRα on the membrane of every cell and OAW28 about 15x10^3 FRα, whereas the other six lines have a number of FRα less than 10x10^3 per cell (Table SI-1).

Supporting Table SI-1. Quantification of the number of FRα in 8 ovarian cancer cell lines, expressed as femto moles per mg of cellular proteins and as the number of receptors per cell. The data are the mean ± S.E.M of three to five experiments performed in duplicate.

Cell lines	Cell surface FRs (fmoles/mg protein)	Cell surface FRs (receptors/cell)
**A2780**	18 ± 1	2.0 x 10^3
**A2780/CP**	13 ± 2	1.1 x 10^3
**2008**	27 ± 5	7.7 x 10^3
**C13***	21 ± 4	7.0 x 10^3
**IGROV-1**	94 ± 4	45.0 x 10^3
**TOV112D**	29 ± 2	8.7 x 10^3
**COV504**	22 ± 12	6.6 x 10^3
**OAW28**	38 ± 6	15.2 x 10^3

LC-MS/MS analysis

1. Sample preparation for LC-MS/MS analysis (FA-LR compartimentalization)

IGROV-1 human ovarian cancer cells were cultured in RPMI medium supplemented with 10% fetal bovine serum (FBS) plus 20mM L-Gln at 37°C with 5% CO_2. RPMI medium was aspirated and replaced by FF-RPMI medium (Folate free RPMI medium) to induce overexpression on the folate receptor on cell surface. After at least 24 hours, FF-RPMI medium was aspirated and replaced by a solution of FA-LR in PBS (5 μM). For the internalization experiments, FA-LR peptide in DMSO (1mg/ml) was diluted with PBS to reach the final concentration of 5 μM. Culture medium was aspirated from cells and FA-LR solution was added to each plate and incubated for 20 min at 37°C, 5% CO_2. The incubation experiments using 5 μM, starting concentration of FA-LR, were then repeated using an incubation temperature of 4°C in order to block the mediated-endocytosis by FR. After that the medium was aspirated, added of ACN (1:1 v/v), centrifuged for 30 min at 14.000 rpm at 4°C, IS was added at the final concentration of 1μg/ml, recentrifuged and then directly injected into LC-MS. Cells, indeed, were washed with ice-cold acid PBS (pH 5.0) for three times, collected by trypsinization and counted. The cell pellets obtained by gentle centrifugation were lysed employing a hypotonic buffer (pH 8.0, 20 mM HEPES, 10 mM KCl, 1,5 mM $MgCl_2$, 1mM EDTA, 250 mM saccarose, 0,1 mM PMSF) containing protease inhibitor cocktail. 100 μl of hypotonic buffer were added every $2x10^6$ cells. Equal aliquots of cell lysate ($2x10^6$ cells/aliquot) were prepared. Three cycles of freeze-thaw were performed. Afterwards, in order to discriminate between uptake into vesicles and cytosol, the samples were centrifuged at 14000 rpm for 30 min at 4 °C. Then ACN was added to the supernatant aspirated from the pellets in order to have 1:1 v/v with the hypotonic buffer solution. While a mixture of ACN:H_2O (50:50, v/v) was added to the pellets. Both supernatants and pellets were centrifuged at 14000 rpm for 30 min at 4°C. Finally, IS at the final concentration of 1μg/ml was added to the pellets and supernatants and they were centrifuged at 14.000 rpm for 25 min. Samples were collected and stored at -20°C. Before LC-MS analysis samples were centrifuged in a

minispin for 10 minutes at 15000 rpm. The amount of peptide in both pellets and supernatants was determined by LC-MS/MS analysis.

2. Sample analysis

FA-LR quantification in cell lysates and cultural medium was performed using an Agilent HP 1200 liquid chromatograph (Agilent technologies, Milan, Italy) consisting of a binary pump as pump 1, an autosampler and a thermostated column compartment. Chromatographic separations were performed on the analytical column (Agilent Poroshell 120 column, 2.1x100mm, 2.7μm particle size). Mobile phase A consisted of 0.1% HCOOH and mobile phase B was ACN. The optimized elution profile was composed of a first isocratic step water (+0.1% HCOOH): ACN 95:5 (v/v) for 0.1 min and then to 41% of ACN over 10 min providing the elution of FA-LR and IS separately from the other interferences contained in the matrix. At 10.1 min ACN raise up to 95% for 5 min to wash the column. Finally, an equilibration step with 95% of aqueous solvent was provided for 4 min in order to restore the initial condition. Flow rate was 0.3 ml/min. Injection volume was 5 μL. An Agilent 6410 triple quadrupole-mass spectrometer (Agilent technologies, Milan, Italy) with an ESI source operating in positive mode was used for detection. The optimized source parameters for MS analysis were: drying gas temperature 350 °C and gas flow 10 L/min, nebulizer gas flow pressure 32 psi and capillary voltage 3500 V. The optimized fragmentor voltage values were 135V for FA-LR and IS. Subsequently, positive ion mass spectra for FA-LR and IS were generated by flow injection analysis in full scan mode at the mass range *m/z* 50–1200. In order to determine the characteristic mass fragments for selected reaction monitoring (SRM) analysis, the product spectra of FA-LR and IS were recorded in full scan mode by varying the offset voltage between 5 and 20 eV. The optimal collision energy values were15 eV for FA-LR and IS. The quantitative analyses were carried out using selected reaction monitoring (SRM) following the reactions: *m/z* 717.2->294.7 as quantifiers, *m/z* 717.2-> 1138.8 as qualifiers for bi-charged FA-LR and *m/z* 478.5->294.6 as quantifiers and *m/z* 478.5-> 896.6 as qualifiers for three charged FA-LR. Transitions for IS were 477.4 - > 409.0 as quantifiers and *m/z* 477.4- > 521. as qualifiers for IS.

3. Method validation of LC-MS/MS

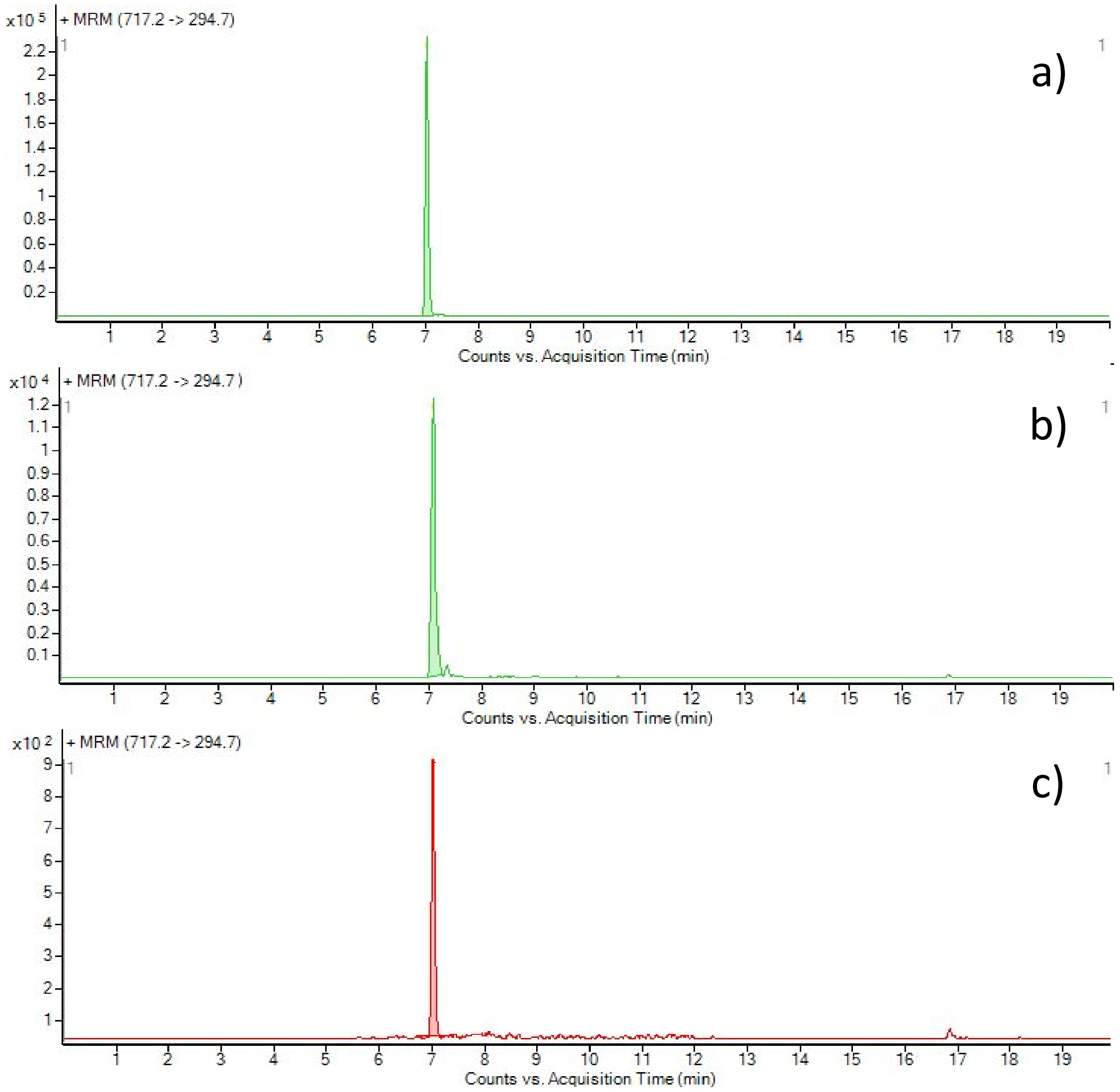

Supporting Figure SI-5. Example of MRM chromatogram obtained analyzing a) starting solution of FA-LR [10µM] b) PBS medium after 20 minutes of incubation at 37°C; c) cell lysates after 20 minutes of incubation at 37°C.

Detection and quantification limits (sensitivity)

Lower limit of quantification, LLOQ, was defined as the concentration at which the quantifier transition of FA-LR yielded a signal to noise ratio of 10 and it was 0.10 µg/ml and 0.12 µg/ml in cell lysate and PBS medium, respectively. Limit of detection (LOD) was estimate based on 3:1 signal-to-noise ratio and it was 0.04 µg/ml in cell lysates and PBS.

Specificity

Typical MRM chromatograms of FA-LR and IS in cell lysate showed no interference peaks from endogenous substances at the retention times of LR and IS. The chromatograms of blank cell lysate also had no interference peaks at the retention times of FA-LR and IS. The specificity results indicate that the method is highly selective for FA-LR analysis.

Calibration curve and Linearity

Calibration curves for FA-LR were performed in cell lysates (0.10 to 8.00 µg ml^{-1}) and in RPMI medium (0.12-10.00 µg ml^{-1}). Five replicates were used to establish the linear calibration equation (y=mx+c) and analyzed using the ratio of analyte peak area over IS peak area after quantitative integration by MassHunter software. Linearity was measured as the coefficient of determination (R^2) measured for five calibration replicates. The acceptance criteria for each back-calculated standard concentration were ±15% deviation from the nominal value except at LOQ, which was set at ±20%. Linearity was excellent over the respective calibration ranges with corresponding correlation coefficient > 0.99. The results are reported in Table SI-2.

Table SI-2. Calibration curves, LLOQ and LOD in cell lysates, DMEM and RPMI medium.

	**Concentration ranges (µg/ml)**	**Regression analysis equations**	**R^2**	**LLOQ**	**LOD**
Cell lysate	0.10 – 8.00	y=10.78x-4.52	0.993	0.10	0.04
RPMI medium	0.12 – 10.00	y=26.66x-5.79	0.994	0.12	0.04

Accuracy and precision

Intraday accuracy and precision were assessed from five replicates of LLOQ and three QC concentration levels (low LQC, middle MQC and high HQC). Interday accuracy and precision were assessed on triplicates for five-day analysis of the tree QC concentration levels. Accuracy of the method is shown as % deviation and was calculate based on the difference between the mean concentration found and concentration added. The % deviation should be within 15% of the actual value, except at the LLOQ, where it should not deviate by more than 20%. Precision was evaluated based on the percentage coefficient of variation (%CV) of the mean concentration found. The precision determined at each concentration level should not exceed 15% of the CV, except for the LLOQ, where it should not exceed 20% of the CV. The accuracy and precision data for cell lysate and RPMI medium are summarized in table SI-3, SI-4. The intraday accuracy and precision showed % deviation ranged from -1.19 to 2.44 % with a %CV from 0.88 to 4.65 % in cell lysates, % deviation ranged from -1.02 to 4.59 % with a %CV from 1.21 to 14.58 % in RPMI medium. The interday accuracy and precision showed % deviation ranged from to -3.18 to -5.44 % with a %CV from 2.32 to 2.98 in cell lysate and % deviation ranged from -7.48 to -3.09 with a %CV from 1.46 to 8.73 % in RPMI medium. These results suggested that the method is accurate and precise.

Table SI-3. Intraday and Interday accuracy and precision in cell lysate

**Concentration added to cell lysate (μg/ml)**	**Intraday**		**Interday**	
	Accuracy (% deviation)	Precision (% CV)	Accuracy (% deviation)	Precision (% CV)
0.50	2.44	3.87	4.22	2.32
3.00	2.20	4.65	5.44	2.98
5.00	-1.19	0.88	-3.18	2.45

Table SI-4. Intraday and Interday accuracy and precision in RPMi medium.

Concentration added to RPMI (µg/ml)	Intraday		Interday	
	Accuracy (% deviation)	Precision (% CV)	Accuracy (% deviation)	Precision (% CV)
0.12	4.59	3.28	-3.09	1.46
5.00	0.26	1.21	-7.48	5.48
10.00	-1.02	14.58	-4.38	8.73

Recovery

The extraction recovery of FA-LR was determined at the LQC, MQC and HQC levels by comparing the peak areas of FA-LR extracted from spiked cell lysate and RPMI samples prepared by spiking FA-LR in blank matrix with the corresponding concentration of authentic standard solutions, experiments were performed in five replicates. The extent of recovery should be consistent, precise and reproducible. The extraction recovery results are shown in table SI-5. The mean recoveries of extraction of FA-LR from cell lysate at three QC concentration levels of 0.50, 3.00 and 5.00 µg/ml were 94.7, 92.3 and 98.0% respectively. The mean recoveries of extraction of FA-LR from RPMI at three QC concentration levels of 0.12, 5.00 and 10.00 µg/ml were 96.00, 96.10 and 98.80% respectively.

Table SI-5. Recovery of LR in cell lysates, DMEM and RPMI medium.

Concentration added to lysates (µg/ml)	% Mean extraction recovery	Concentration added (µg/ml) RPMI	% Mean extraction recovery
0.50	94.70	0.12	96.00
3.00	92.30	5.00	96.10
5.00	98.00	10.00	98.80

Matrix effect

The matrix effect was evaluated by determining the matrix factor (MF) and the IS-normalized MF from cells lysate and RPMI. The determination was performed at the LQC, MQC and HQC levels in triplicate for each matrix. The MF of FA-LR was calculated by comparing the peak area of FA-LR extracted from samples prepared by spiking FA-LR in cell lysate and RPMI with the corresponding concentration of the authentic solution of FA-LR prepared in PBS. The MF of IS was determined and calculated in a similar manner. The IS-normalized MF is the ratio between the MF of FA-LR and that of IS. The %CV of the IS-normalized MF from the three different blank matrixes should not exceed 15%. The effects of cell lysate and RPMI matrix on the analysis of FA-LR are summarized in Table SI5. The %CVs of IS-normalized MF in cell lysate were 9.26, 9.70 and 10.00 for LQC, MQC and HQC, respectively. The %CVs of IS-normalized MF in RPMI medium 4.63, 7.00 and 8.67 for LQC, MQC and HQC, respectively. The %CV<15% for LQC, MQC and HQC suggest that ion suppression or enhancement from plasma matrix was negligible in the assay.

Table SI-6. Matrix effect of FA-LR in cell lysates and RPMI medium at low, medium and high concentration.

**Concentration added to lysates (µg/ml)**	**Mean IS normalized factor (%CV)**	**Concentration added (µg/ml) RPMI**	**Mean IS normalized factor (%CV)**
0.50	73.79 (9.26)	40.30	95.10 (4.63)
3.00	175.14 (9.70)	17.64	101.62 (7.00)
5.00	214.66 (10.00)	39.88	114.73 (8.67)

Fluorescence spectra of a lysate of IGROV1.

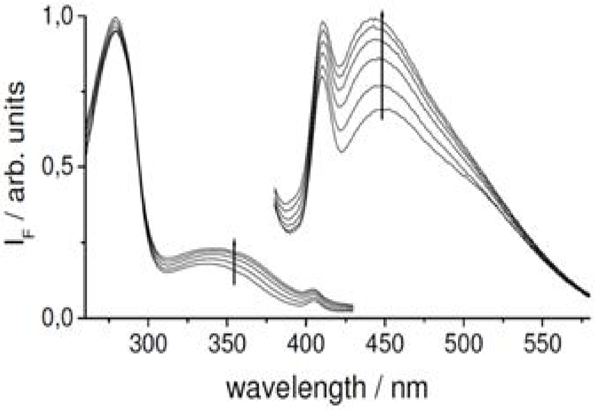

Supporting Figure SI-6. Pteroate excitation (λ_{em} = 450 nm) and emission (λ_{exc} = 360 nm) fluorescence spectra of a lysate of IGROV1 cells treated with FA-LR (see text). The arrows indicate the spectral evolution upon subsequent emission-excitation acquisition cycles. Data are normalized to the maxima of the final spectra for ease of presentation.

Study of FA-LR concentration changes with time using Orbitrap Q-Exactive™ mass spectrometer.

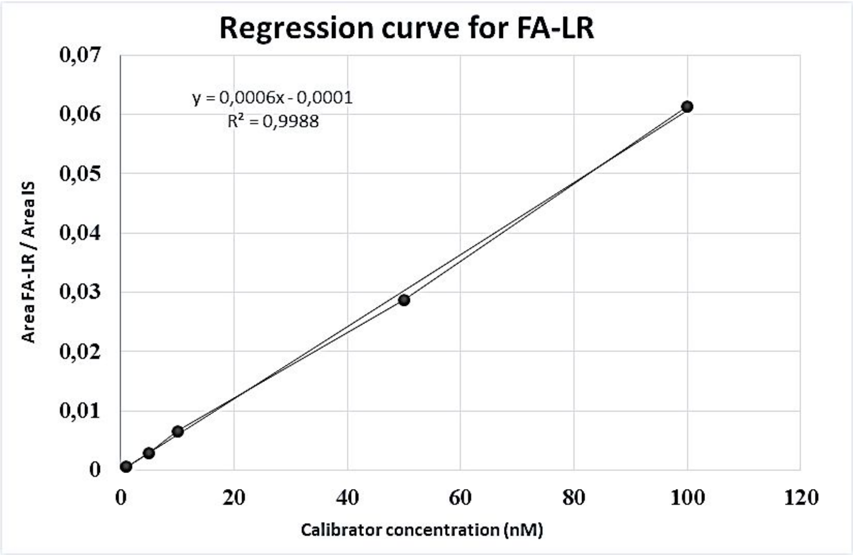

Supporting Figure SI-7. Regression curve for FA-LR concentration changes with time after FA-LR administration. Regression equation y = 0.0006x – 0.0001 $R^2 = 0.9988$. Data obtained with Orbitrap Q-Exactive™ mass spectrometer.

Table SI-7. Concentration of FA-LR per single cell after different time from the exposure for 20 min to FA-LR.

**Time lapse**	**Entire sample (nM)**	**Single cell (µM)**
20min	106.13	18.62
40min	83.37	15.97
1hr	68.89	15.94
2hrs	65.06	12.05
4hrs	62.92	8.6

The concentration of FA-LR per single cell after different time from the exposure for 20 min to FA-LR, was determined using the below reported conversion formula:

$$REAL\ CONCENTRATION = \frac{interpolated\ conc.\ x\ 500\mu L\ (final\ Vol)}{1.2x10^{-12}\ (cell\ Vol)x\ cell\ number}$$

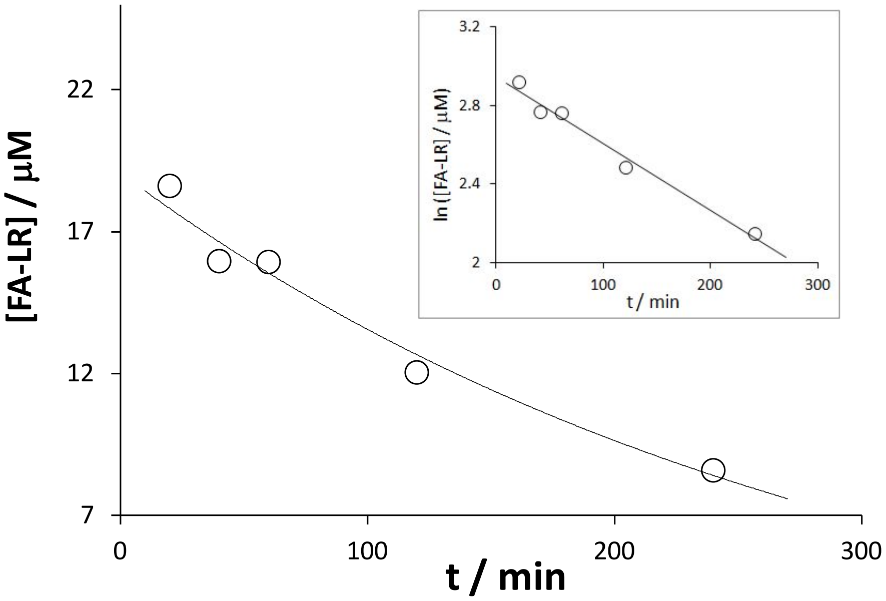

Supporting Figure SI-8. Time-course of the cytosolic FA-LR concentration in C13* cells. Inset: first-order analysis.

Cell survival of human ovarian cancer cell lines (showing low FRα expression) treated with peptide LR and its conjugate alone or transfected into cells by means of peptide delivery system.

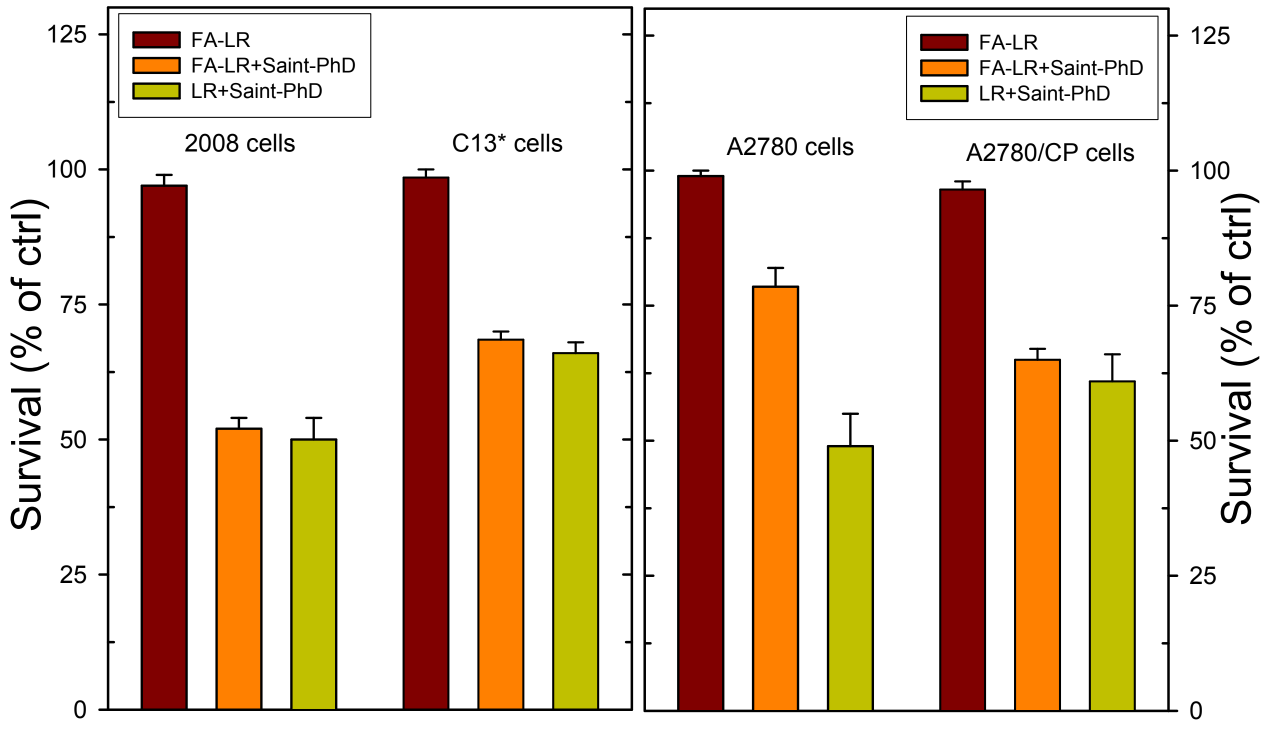

Supporting Figure SI-9. Cell survival of human ovarian cancer cell lines (showing low FRα expression) treated with peptide LR and its conjugate alone or transfected into cells by means of peptide delivery system. Cell lines were treated for 72 hours with equal amounts (5 μM) of FA-LR (alone and with SAINT-PhD) and LR. The bars represent the mean ± S.E.M of three experiments performed in duplicate.

To evaluate the effect on cell growth of low FR expressing cells, the bioconjugate FA-LR was administered as such to exploit the FR-mediated endocytosis, or transfected by means of a specific peptide delivery system, the SAINT-PhD (Synvolux Therapeutics, NL), and comparing it to the peptide LR alone, as a control. 24 hrs after seeding in complete RPMI medium, 2008 and A2780 cisplatin-sensitive cell lines, and their resistant counterparts, C13* and A2780/CP cells, were grown in FF-RPMI medium for 24 hr to maximize the externalization of FR on cell surface. Then, cell lines were treated for 72 hours with 5 μM of FA-LR alone or transfected with SAINT-PhD loaded with the

same amount (5 μM) of FA-LR or LR. At this time point, cell survival was evaluated by crystal violet dye staining. The incorporated dye was solubilized in acidic isopropanol and determined spectrophotometrically with a multiplate reader (TecanGenios Pro with Magellan 6 software) at 540 nm. The extracted dye was proportional to the cellular biomass.

As it appears in Fig. SI-9, these cell lines showing low FR on their surface, were able to almost completely survive to FA-LR treatment; on the contrary, when both FA-LR and LR were transfected by means of the delivery system, which allow them to reach intracellular concentration higher than those obtained by the internalization through the few FR, cell survival was reduced by 25-50 %, depending on cell line.

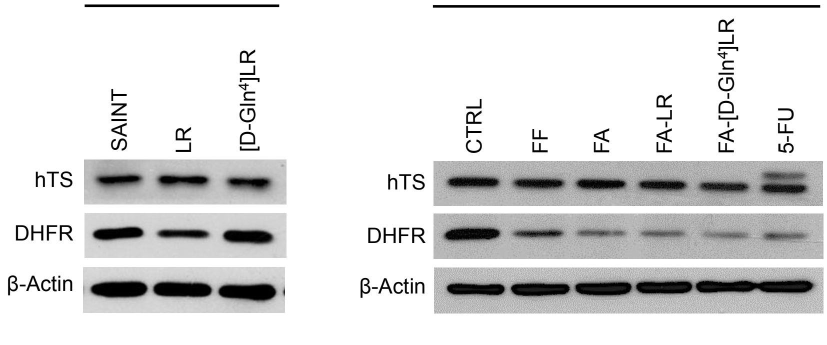

Supporting Figure SI-10. Immunoblot analysis of hTS and DHFR protein levels in IGROV-1 cells. hTS and DHFR protein levels in cells treated with LR and [D-Gln^4]LR by using a peptide delivery system (SAINT-PhD, left) and with 1.25 μM FA, FA-LR, FA-[D-Gln^4]LR and 5-FU for 48 hours (right). Cells were plated in complete medium containing 10% heat-inactivated FBS and after 24 hours, in FF-medium, except for the control sample (CTRL). After additional 24 hours, cells were treated with FA, bioconjugates and 5-FU.

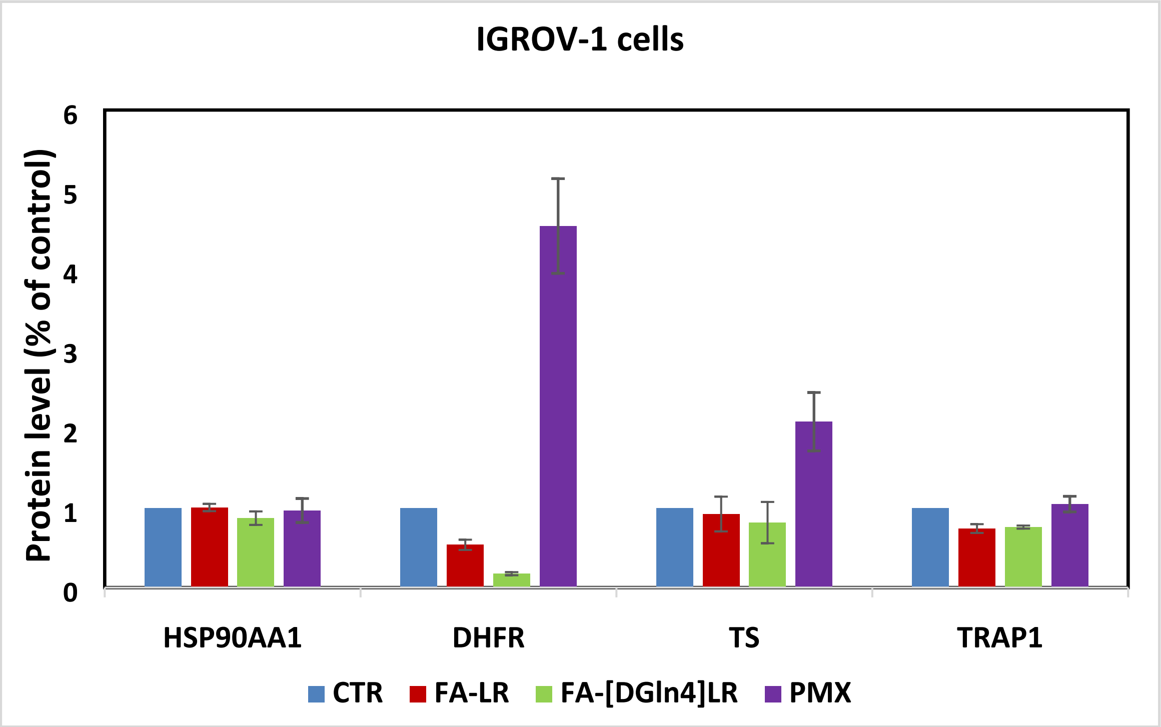

Supporting Figure SI-11. Densitometric scanning analysis of the blots related to hTS, DHFR, HSP90AA1 and TRAP1 protein levels in IGROV-1 cells treated with FA-LR, [D-Gln^4]LR and PMX for 48 hours. Cells were plated in complete medium containing 10% heat-inactivated FBS and after 24 hours, in FF-medium, except for the control sample (CTRL). After additional 24 hours, cells were treated with bioconjugates and PMX.

Table SI-8. Effects of drug combinations on A2780 and IGROV-1 cell growth. The nature of the combination was obtained by evaluation of SQ values after treatment of cells with drugs alone and in combination.

Drug combination	% Growth inhibition[a]	Synergism quotient	% Growth inhibition[a]	Synergism quotient
	**A2780 cells**		**IGROV-1 cells**	
**FA-LR 250 nM plus**	12.8		14.2	
**cDDP 1µM**	37.9 / 12.8+37.3	0.75	63.2 / 14.2+40.3	1.16
**cDDP 2µM**	47.6 / 12.8+49.1	0.77	59.6 / 14.2+44.9	1.01
**cDDP 5µM**	87.9 / 12.8+78.6	0.96	54.6 / 14.2+48.8	0.86
**FA-LR 250 nM plus**				
**5FU 7.5µM**	25.1 / 12.8+15.7	0.88	46.3 / 14.2+29.8	1.05
**5FU 10µM**	53.5 / 12.8+45.3	0.93	47.5 / 14.2+28.4	1.11
**5FU 15µM**	49.6 / 12.8+34.5	1.06	45.9 / 14.2+36.5	0.91
**FA-LR 250 nM plus**				
**RTX 10nM**	62.9 / 12.8+56.8	0.90	49.6 / 14.2+34.5	1.10
**RTX 20nM**	64.8 / 12.8+59.9	0.89	40.5 / 14.2+29.5	0.92

[a] Data are reported as (combination inhibition)/(inhibition of single compounds).

In Table SI-8, data are expressed as average values percentage of growth inhibition in reference to the growth of untreated control cells of duplicate cell counts on three or more separate experiments and indicate the inhibition of drug combination divided by the sum of the inhibition of single drug to obtain the values of synergism quotient (SQ). A quotient of > 1 indicates a synergistic effect, between 0.9 and 1.1 indicates an additive effect, while a quotient of < 1 indicates an antagonistic effect.

References

1. Carosati E, Tochowicz A, Marverti G, Guaitoli G, Benedetti P, Ferrari S, Stroud RM, Finer-Moore J, Luciani R, Farina D, Cruciani G, Costi MP. Inhibitor of ovarian cancer cells growth by virtual screening: a new thiazole derivative targeting human thymidylate synthase. *J Med Chem.* 2012, *55*, 10272-10276.

2. Reddy, J.A., Haneline, L.S., Srour, E.F., Antony, A.C., Clapp, D.W., and Low, P.S. (1999) Expression and functional characterization of the betaisoform of the folate receptor on CD34(+) cells. *Blood 93*, 3940–3948.
